# Integrative genomics would strengthen AMR understanding through ONE health approach

**DOI:** 10.1016/j.heliyon.2024.e34719

**Published:** 2024-07-17

**Authors:** Chinky Shiu Chen Liu, Rajesh Pandey

**Affiliations:** a Division of Immunology and Infectious Disease Biology, INtegrative GENomics of HOst-PathogEn (INGEN-HOPE) Laboratory, CSIR-Institute of Genomics and Integrative Biology (CSIR-IGIB), Mall Road, Delhi, 110007, India; b Academy of Scientific and Innovative Research (AcSIR), Ghaziabad, 201002, India

**Keywords:** Genomics, AMR, Pathogen evolution, Infectious diseases, ONE health

## Abstract

Emergence of drug-induced antimicrobial resistance (AMR) forms a crippling health and economic crisis worldwide, causing high mortality from otherwise treatable diseases and infections. Next Generation Sequencing (NGS) has significantly augmented detection of culture independent microbes, potential AMR in pathogens and elucidation of mechanisms underlying it. Here, we review recent findings of AMR evolution in pathogens aided by integrated genomic investigation strategies inclusive of bacteria, virus, fungi and AMR alleles. While AMR monitoring is dominated by data from hospital-related infections, we review genomic surveillance of both biotic and abiotic components involved in global AMR emergence and persistence. Identification of pathogen-intrinsic as well as environmental and/or host factors through robust genomics/bioinformatics, along with monitoring of type and frequency of antibiotic usage will greatly facilitate prediction of regional and global patterns of AMR evolution. Genomics-enabled AMR prediction and surveillance will be crucial - in shaping health and economic policies within the One Health framework to combat this global concern.

## 1 Introduction

**Fig. 1:**
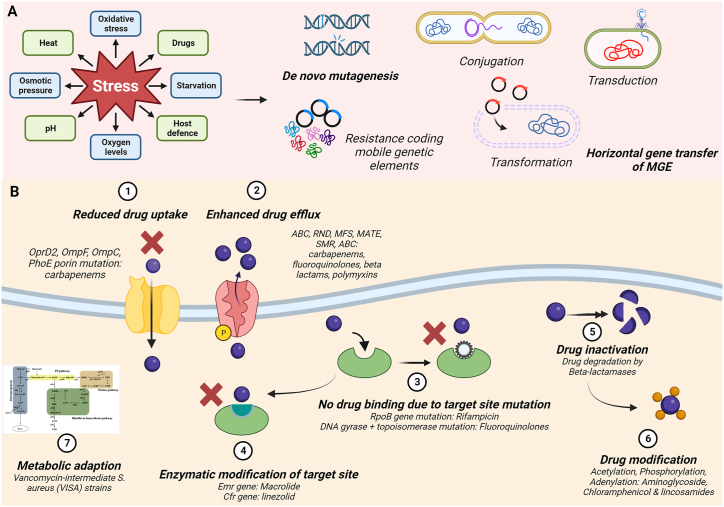
**Mechanisms of antimicrobial resistance. A.** Environmental stress factors trigger acquisition of novel mutations by pathogens that allow pathogen adaptation and survival under stressed conditions. Acquisition of mutations can be due to *de novo*-mutagenesis or acquisition of mobile genetic elements (MGEs) including plasmids and transposons through horizontal gene transfer (HGT) processes like transformation, conjugation and transduction. *De novo* mutations in genes involved in drug binding or response as well acquisition of drug resistance-encoding elements through HGT will result in generation and propagation of subpopulation of microbes that exhibit AMR. **B.** Mutations involved in emergence of drug resistance can be generally categorized as those that target drug receptors, resulting either in their downregulation or drug binding site mutational or enzymatic alterations, both of which result in reduced drug uptake. For e.g., Carbapenem resistance in gram-negative bacteria show predominance of mutations in outer membrane protein channels like OprD2, OmpF, OmpC, PhoE porin mutations. This results in reduction in cellular permeability to nutrients, amino acids and peptides including drugs causing AMR development. Mutations that alter the drug binding site in the microbe also hinder effective drug uptake. These include rifampicin resistance associated with mutations in its target protein binding site of RNA polymerase β subunit and mutations in the GyrA and ParC subunits of gyrase and topoisomerase IV that prevent drug binding to the DNA-enzyme complex, resulting in resistance. Enzymatic modification of the drug target site also leads to AMR. These include acquisiton/upregulation of erythromycin resistance rRNA methylase (erm) and chloramphenicol–florfenicol resistance (cfr) gene coding for RNA methyltransferases modifying bacterial 23S rRNA, conferring resistance to macrolide, lincosamide, and streptogramin, oxazolidinones and pleuromutilins. AMR also involves upregulation of cation channels or drug efflux pumps that enhance extracellular secretion of drug molecules, thereby preventing their action on molecular targets of the pathogen. Other mechanisms include enzymatic modifications or inactivation of the drug, which prevents its antimicrobial efficacy in the host despite cellular uptake. These include degradation of β-lactam drugs like penicillin and cephalosporins, aminoglycosides by microbial hydrolysis. Enzymatic modification of drugs like acetylation (aminoglycosides, streptogramins), phosphorylation (aminoglycosides, chloramphenicol), and adenylation (aminoglycosides, lincosamides) produces steric hindrance effectively reducing drug-target binding avidity. Many drug resistance mutations also produce metabolic adaptations of the microbe. For e.g., Vancomycin-intermediate *Staphylococcus aureus* (VISA) have the capacity to use alternate carbon sources trough acetogenesis, pentose phosphate pathway, purine biosynthesis pathway, wall teichoic acid and peptidoglycan precursor biosynthesis pathway due to reduced tricarboxylic acid (TCA) activity.

Antimicrobial resistance (AMR) is a rising public and global health emergency that poses the significant threat of emergence of drug-resistant microbes and associated diseases [1]. Although being a natural consequence of pathogen evolution, evolution and spread of AMR has become a global health emergency [2]. Microbes naturally undergo genetic evolution as a result of acquisition of external genetic elements or intrinsic mutations to optimize their survival and fitness under unfavorable habitat conditions or selection pressures **(Box 1)** [3]. Unregulated use of antimicrobial agents including antimicrobial drugs, disinfectants, heavy metals and biocides has greatly spurred this natural evolution by allowing microbes to thrive under these high selection conditions **(Box 2)**. The extensive utilization of antimicrobial agents in hospitals, healthcare facilities, agriculture, industries, and livestock, coupled with the spillover of these agents into environmental ecosystems, has resulted in widespread dissemination of AMR across the ecosystems and concomitant surge of multi-drug resistant infections [3,4]. This alarming trend significantly contributes to the high mortality rates associated with infection-related illnesses in humans. Prevalence of drug resistance in infectious and non-infectious diseases have been described in Box 3. A brief description of the cellular and molecular mechanisms underlying AMR phenomenon has been provided in Box 4 and Fig. 1. Current strategies to combat existing drug-induced resistance in microbes have been described in Box 5.

The Global Health Security Agenda (GHSA) was launched in 2014 to foster collaboration between the US, partner nations and international organizations that aimed at boosting global health security by aiding measures to prevent and contain infectious diseases [2]. AMR remains as one of the priority objectives of GHSA under the Medicines, Technologies, and Pharmaceutical Services (MTaPS) Program operating in 13 target countries [5].

In addition to frequency of usage of antimicrobial agents, AMR spread is contingent upon critical socioeconomic factors. Sanitation and waste management, pollution, nutritional status and humans and animal migration across habitats, are critical variables that determine rates of emergence and spread of AMR [6]. Considering these multifaceted parameters, AMR becomes an ideal candidate for the One Health approach, enabling surveillance and policy development across multiple sectors simultaneously.

Given that the genetic evolution of microbes under different selection pressures drives AMR, the incorporation of technologies facilitating genomic surveillance of these microbes across a range of environmental habitats should be intricately integrated into the One Health strategy [6,7]. This integration is essential for effectively monitoring and predicting the rates of mobilization and persistence of resistance-causing genetic elements or mutations across various clinical, industrial, agricultural and veterinary sectors.

Here, we review the latest findings and advancements in the domain of genomic surveillance which have significantly contributed to the One Health strategy in combating AMR.

### 1.1 One health strategy to combat antimicrobial resistance

**Fig. 2:**
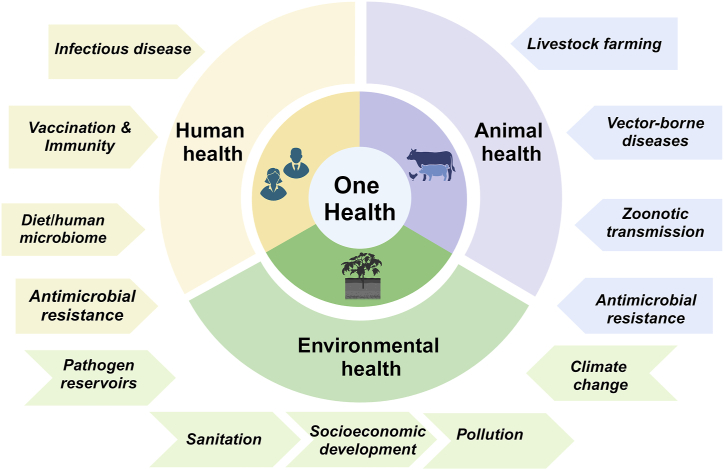
**The One Health strategy of monitoring global health.** One Health encompasses a unified approach that merges inter-domain efforts focused on improving the interconnected well-being of humans, animals, and the environment, at both regional and global levels. Initially conceived to monitor the origins and transmission of infectious diseases, its present goals encompass ongoing surveillance of circulating infectious agents or antimicrobial-resistant pathogens and their cross-species and cross-border transmission. It rigorously assesses the influence of biotic, abiotic, and environmental factors, as well as socioeconomic variables, which potentially shape the epidemiology of diseases.

The concept of ‘One Health’ entails a cohesive strategy that integrates interdisciplinary endeavors aimed at enhancing the three pillars of global health - humans, animals and the environment, on both regional and global scales [6,7]. This approach recognizes the intricate interconnection between health of humans, animals, ecosystems and their significant influence on the emergence and spread of diseases (Fig. 2). The significant human fatalities and economic setbacks resulting from the highly pathogenic avian influenza (HPAI) outbreak caused by subtype Influenza A (H5N1), leading to emergence of severe acute respiratory disease (SARS) across Asia, Africa, and Europe in 2003 prompted a global response involving comprehensive international efforts to curb the pandemic. As a consequence, the world was confronted for the first time with the hard realization of its inadequacy in combating a virus that was already wreaking havoc on the poultry industry. The One Health initiative task force was established back in 2006 to facilitate collaborative efforts between diverse public sectors including government agencies, academic institution and health science professionals [8,9]. Its objective was regional and global surveillance and risks assessment of zoonotic disease transmission with subsequent implementation of measures for their prevention and treatments. This initiative fostered collaboration between major global organizations including the World Health Organization, FAO (United Nations Food and Agriculture Organization), the World Organization for Animal Health (OIE), UNICEF (United Nations Children's Fund), along with the World Bank and UNSIC (United Nations System Influenza Coordinator). Together, they authored a landmark document titled “Contributing to One World, One Health: a Strategic Framework for Reducing Risks of Infectious Diseases at the Animal–Human–Ecosystems Interface,” which laid the foundation for the ‘One World, One Health’ strategy [10,11]. The One World, One Health strategic framework was founded to monitor emerging infectious diseases (EID) at the ‘animal-human-ecosystem interface’ to prevent or minimize global impacts of potential pandemics and/or epidemics. In 2020, the World Health Organization (WHO) in close collaboration with Food and Agriculture Organization of the United Nations (FAO), the United Nations Environment Programme (UNEP) and the World Organization for Animal Health (WOAH) formed the interdisciplinary One Health High-Level Expert Panel (OHHLEP) to fuel interdisciplinary collaboration and implement executive measures of the One World, One Health strategy (One Health High-Level Expert Panel [12]. Currently, the One Health strategy encompasses global health crisis including AMR, zoonotic infectious diseases, vector-borne disease, contaminated foodborne diseases and environmental concerns like pollution and climate change.

Robinson at el. (2016) [13], aptly proposed that AMR should be regarded as a One Health concern due to its global emergence and dissemination which can be attributed to the transmission of resistant microbes across species and ecosystems. The causal relationship between spread of resistance and unregulated use of antibiotics for human and livestock treatments, and resulting prevalence of antibiotics and associated resistance in the local environment underscores the importance of adopting One World, One Health approach for combating AMR [6,14]. Prolonged use of antibiotics can cause fixation of resistance-conferring genes in the resident microbe which can make way into the food chain through contaminated food and water source, or through fecal matter in the environment. This results in widespread dissemination of AMR through environmental reservoirs consisting of contaminated food, water, soil, hospital, industrial and farm wastes. It has however, been observed that duration and/or frequency use of antibiotics does not always strongly correlate with levels of AMR within and across nations. Other socioeconomics factors like public health, nutritional status, development, access to antibiotics, sanitation, human and animal migration and trade also have major impacts on spread of AMR [[15], [16], [17]]. All these parameters can be systematically monitored within the framework of the One World, One Health approach.

A critical aspect of One World, One Health framework in combating AMR involves constant regional and global monitoring of abundance and persistence of resistant microbial populations harboring resistant-causing genetic features, across various environmental niches. This is essential in monitoring the rates of emergence and evolution of resistant microbial population and their dissemination through ecosystems. Genomic surveillance of microbial populations residing in different ecological niches can thus, greatly facilitate prediction of AMR emergence and enable implementation of proactive measures for its prevention.

### 1.2 Integration of genomic surveillance within the one health strategy

Genomic evaluation and assessment of microbial populations should be one of the primary focusses of the One Health framework of AMR surveillance [18,19]. As described before, a plethora of studies have highlighted genetic evolution of microbes as drivers of AMR development. Identification of genetic determinants that confer resistance, estimation of their frequencies in various microbial populations, along with elucidation of their mobilization and transmission dynamics, can facilitate prediction of patterns of AMR spread. The next few sections delve into current developments and limitations in the aforementioned aspects of genomics-enabled AMR surveillance. This includes a comprehensive examination of key developments in NGS-based technologies for detecting genetic determinants of AMR in both pathogenic and commensal microbes, as well as monitoring their global clonal dissemination, and spread across different ecological niches (such as water, air, soil, animals, and food) spanning diverse geographical habitats. By employing phylodynamic-based modelling approaches and integrating machine learning strategies for predicting AMR trends, efforts can be directed towards effectively curating unified and standardized AMR genomic databases and interpretation guidelines that can be universally adopted by healthcare workers, researchers, and policymakers within the One Health strategy of combating AMR.

### 1.3 Detection of antimicrobial resistance

Development of AMR is largely predictable in a bacterial population where class of antibiotic and its frequency of use positively correlates with its resistance in the exposed bacterial population [20,21]. Various parameters influence emergence of drug resistance in a microbial population. They include mode of pathogen transmission, rate and duration of infection, ability to adapt to host conditions (pathogen fitness) and cross-resistance to other drugs [20,21].

### 1.4 Conventional approaches of AMR identification

**Fig. 3:**
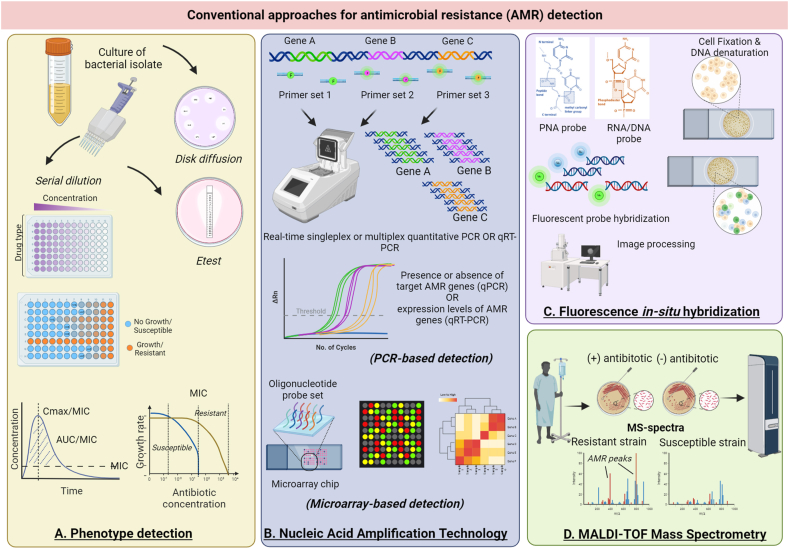
**Conventional approaches for antimicrobial resistance (AMR) detection.** A. Phenotypic detection. Conventional approaches to determine antimicrobial resistance to putative drugs involved in-vitro screening of known microbial species against a panel of potentially resistant or susceptible drugs. Culture-based conventional antibiotic susceptibility testing (AST) includes disk diffusion test (also known as Kirby–Bauer test). Bacteria isolated from patients is grown on agar plates and antibiotic-adsorbed papers are disks are applied on to the plates and incubated. Depending on bacteriostatic and bactericidal activity of the applied antibiotics, a zone of bacterial growth inhibition will be observed in regions where the disks were placed. Depending on the size zones of inhibition, antibiotic susceptibility can be estimated semi-quantitatively as susceptible, moderately resistant and strongly resistant. Other quantitative estimations where minimum inhibitory concentration (MIC) can be calculated include serial dilution and Etest methods. Etest method (Epsilometer test) is based on the principle of disk diffusion method with the difference being that it is performed on agar plates with antibiotic strips of a wide range of predefined gradients of concentrations. This allows quantitative estimation of MIC values. Similarly, assays involving serial dilution of a panel of antibiotics can also be used to obtain correlation between bacterial growth rates and antibiotic type and concentrations. Strongly resistant, intermediately resistant or susceptible strains were identified on the basis of the minimum inhibitory concentration (MIC) calculated from drug dose response-killing assays. **B.** Nucleic acid amplification technology (NAAT)-based detection. NAAT technology employs PCR-based amplification and detection of targeted AMR elements. Using defined primer sets against known genetic drivers or mediators of AMR, this method can be used to detect presence or absence of such elements in the sample-of-interest. This method can utilize either singleplex, duplex or multiplex PCR detection depending on use of probe sets against a single, double or more than 2 AMR gene-of-interest. Certain multiplex assays allow simultaneous detection of a panel of up to 28 AMR markers against 9 classes of antibiotics from 26 pathogens. While simple presence or absence of AMR elements does not accurately reflect their role in AMR resistance profiles, quantitative real-time PCR (qRT-PCR) can be used to assess expression levels of these AMR elements and their potential contribution towards resistant phenotype. An improvement over PCR-based detection is the use of DNA microarrays where a much larger set of fluorescent oligonucleotide probes against potential AMR elements. Immobilization of these probes on a solid matrix enables simultaneous detection of multiple AMR sequences from different pathogens. This facilitates recognition of multiple mechanisms of AMR and their variants in a single assay. **C.** Fluorescence *in-situ* hybridization (FISH). Hybridization-based techniques including quantitative FISH (Q-FISH) enables rapid screening for AMR makers in samples. New generation Q-FISH employs use of synthetic probes instead of conventional fluorescently-labeled RNA/DNA probes. These synthetic probes are called peptide nucleic acid (PNA) oligonucleotides and are DNA mimics. They have the added benefit of having a neutral DNA backbone and can thus, bind with higher efficiency to target DNA compared to conventional DNA/RNA probes having charged backbone. **D.** Mass spectrometry (MS) analyses of AMR profiles. Matrix-assisted laser desorption ionization-time of flight mass spectrometry (MALDI-TOF-MS) is an efficient rapid, low-cost technique of direct characterization of microbial protein products (proteomics) or metabolites (metabolomics). MS is particularly useful for elucidation of mechanisms of resistance including antibiotic modification (hydrolysis of β-lactams antibiotics or acetylation of fluoroquinolones) as well detection of AMR marker proteins.

Identification of resistant strains traditionally relied on in-vitro antimicrobial susceptibility tests with cultures of bacteria exposed to target drugs (Fig. 3A). Culture-based detection of antimicrobial susceptibility relies on estimation of minimum inhibitory concentrations (MIC) values [22]. This technique however, can be limited to those strains which can be cultured ex-vivo [22,23]. This is severely limiting as environmental microbiologists have estimated that about 70 % of bacteria are unculturable in laboratories [24]. Moreover, this approach does not provide insight into the mechanism of resistance. Molecular typing allows rapid culture-dependent/independent detection of antibiotic susceptibility along with identification of genetic drivers of resistance [25,26]. PCR-based detection allows rapid screening of AMR using primers against known AMR genes (Fig. 3B). Real time multiplex PCR enables phenotypic characterization of resistant microbes by possessing an added advantage of simultaneous screening of a panel of AMR genes from a diverse range of pathogens, known to drive resistance against several classes of antibiotics. While PCR offers rapid detection of AMR, it requires prior culture of microbes and does not provide any insight into the mechanism of resistance. DNA microarrays serve as a marked improvement over PCR-based detection since it allows screening of a much larger panel of AMR genes from a diverse range of pathogens in a single assay (Fig. 3B). While molecular-based detection of AMR is rapid, accurate and sensitive, they are severely limited by the fact that they can identify presence or absence of known AMR-driving genes and mutations. They do not allow detection of novel mechanisms leading to resistance and thus, enables incomplete characterization of drug-resistance phenotype of microbes. Other approaches for AMR detection involves hybridization assays including quantitative fluorescent *in-situ* hybridization as well as mass spectrometry to analyze proteomic or metabolic profiles associated to resistance described in Fig. 3C & D, respectively.

### 1.5 Next generation sequencing technologies

**Fig. 4:**
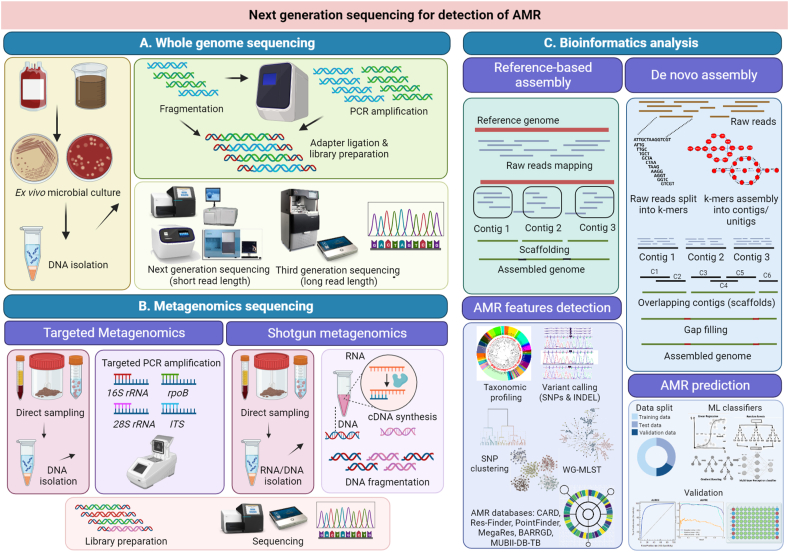
**Next generation sequencing for detection and prediction of AMR.** Next generation sequencing for detection and prediction of AMR. **A.** Microbial NGS can be performed as whole genome sequencing (WGS) or metagenomic sequencing. WGS involves complete microbial genome sequencing but requires prior cultivation and amplification of microbial isolates from clinical samples so as to provide sufficient samples for appropriate sequencing depth and coverage. DNA isolated from cultivated samples can also be further amplified by PCR. DNA and/or PCR products are subjected to fragmentation, tagging and adaptor ligation (library preparation) and sequenced. Second generation sequencing platforms like Illumina, Solexa, Roche 454 and Ion Torrent typically produce short sequencing read lengths of up to 500bp. Third generation sequencing platforms include Oxford nanopore and Pacific Bio, where read lengths can exceed 10 kb. While shorter read lengths are difficult to assemble, they exhibit high accuracy rates as opposed to long read lengths. **B.** Metagenomic sequencing involves direct sequencing of DNA isolated from the entire microbial community in the primary sample without prior cultivation. Metagenomic sequencing can be either targeted where primers against specific DNA markers like 16sRNA, rpoB, 28SrRNA and nuclear ribosomal internal transcribed spacer (ITS, for fungal isolates) region. Shotgun sequencing is similar to WGS where the entire genome is sequenced, albeit at lower sequencing depth. Despite requiring a complex pipeline for assembly and analysis, shotgun sequencing can provide profiles of all types of microorganisms present in the primary sample. Shotgun sequencing also provides a higher taxonomic resolution at the species level. While targeted metagenomics cannot directly profile gene functions, it can be used to predict functional profiles when used with certain tools (PICRUSt package). Shotgun sequencing overcomes this barrier but comes with limitations of higher costs, complex analysis pipeline, and requirement of higher computational power and time. **C.** Bioinformatics pipeline for AMR detection and prediction broadly involves the following steps. *Genome Assembly:* Reads obtained after sequencing subjected to filtering by removing low quality reads (failed base calling) and trimming or removal of adaptor sequences. Construction of the genome from filtered reads can be performed by 2 methods. Reference-based genome assembly requires mapping the reads to a reference genome. While being less computationally intensive, the assembly will be highly biased to the reference genome being used. De novo assembly does not use any reference genome. The reads are clustered on the basis of overlapping sequences to form contigs which are further aligned to form scaffolds. The gaps between scaffolds are carefully filled by other non-overlapping reads to form the complete assembled genome. The processes of scaffolding and gap-filling are subjected to numerous reiterations so as to enhance accuracy and efficiency. The most common approach for assembly of shorts reads is the de Bruijn graph where reads are converted into overlapping k-mers. The de Bruijn graph constructs an optimal directed path for genome assembly based on overlapping k-mers. K-mers are also employed in sequencing error corrections before or after genome assembly. Uniform amplification and sequencing depth will result in a normal distribution of k-mer depth. k-mer depth show an exponential decrease during sequencing errors, so k-mers below a threshold depth can be excluded during genome assembly. *Detection of AMR features:* After genome assembly, AMR features can be identified. AMR features consists of coding genes or non-coding genetic elements know to drive or mediate resistance to antibiotics. Variant calling including identification of SNPs (single nucleotide polymorphisms) and INDELs (insertion-deletion) mutations facilitates construction of the genetic profile associated with resistance phenotype. NGS facilitates whole genome multi-locus sequence typing (WG-MLST). WH-MLST enables AMR profiling based on allelic variations, recombinations and INDELs at multiple positions. It can be used to decipher evolutionary relationships between various clinical isolates of outbreak strains. Identified genomic features can be classified as AMR elements by comparing with AMR databases like The Comprehensive Antibiotic Resistance Database (CARD), ResFinder, PointFinder, MegaRes, Bacterial Antimicrobial Resistance Reference Gene Database (BARRGD) and MUBII-TB-DB (particularly for AMR in *Mycobacterium tuberculosis*). *AMR prediction:* Prediction of AMR profiles from NGS data requires use of machine learning (ML). Various ML classifiers including linear regression, random forest, LightGBM or XGBOOST gradient boosting and Neural Network - Multi-layer Perceptron Classifier (MLP classifier) are used to predict antibiotic resistant genome profiles. Classifier performance can be validated using estimations of sensitivity and specificity- AUROC (Area Under Receiver operating characteristic Curve) and AUCPR (Area Under Precision-Recall Curve). AU-ROC is measured as correlation between true-positive rate (sensitivity) and false-positive rate (specificity) while AUCPR is measured as correlation between precision and recall. Experimental validation through AST and genetic screens can further validate predicted genomic features associated with AMR.

NGS technologies coupled with machine learning have greatly enhanced understanding of the temporal and spatial evolution of pathogen strains and its genetic drivers or regulators of AMR (Fig. 4) [[27], [28], [29], [30]]. Next generation sequencing involves both metagenomic sequencing of all bacterial species from primary samples or whole genome sequencing (WGS) from cultured bacterial species of interest [31,32]. While metagenomic sequencing allows direct taxonomic characterization of microbial diversity either through targeted bacterial 16S rRNA gene sequencing or whole genome shotgun sequencing, these techniques are burdened with high costs, shorter reads and low sequencing depth of individual microbial species in the primary samples [32]. This often results in considerable loss of genome coverage along with low abundance of microbial sequences and large background noise in complex metagenomic datasets. Reducing background data is currently being achieved by employing various capture techniques for enrichment of target DNA. These include poly (A) tail selection, CRISPR and hybridization arrays and primer-based amplification. Techniques like CRISPR and riboZero (ribosomal RNA depletion) can also be used to reduce background non-microbial DNA [19,[33], [34], [35]]. One of the major disadvantages of shorter reads of shotgun metagenomics (150-300bp) is loss of data of genetic context of resistance-conferring DNA elements or complex resistance regions (CRR) [19]. This is particularly critical as the genetic context of resistance genes has been shown to significantly influence the acquisition of AMR genes and their transmission across microbial and host populations. Therefore, metagenomic sequencing does not facilitate optimal characterization of antibiotic resistance profiles of individual microbial species/isolates in the primary samples. They are however, useful for study of difficult-to-culture microbes like *Mycobacterium tuberculosis.* WGS, on the other hand, is frequently used to delineate the complete resistance phenotype of the microbe-of-interest [30]. Its rapidity, high sensitivity (larger depth of sequencing) and accuracy facilitates simultaneous screening and identification of multiple genetic loci which are drivers or correlates of AMR. WGS, however, fails to capture the microbial population diversity of samples in their ecological niche and thus, may not be very effective at monitoring AMR genomic surveillance. Recent advancements in long-read metagenomics and assemblers has enabled complete characterization of resistance-harboring plasmid sequences and genomic regions flanking AMR genes from complex human gut microbiome samples [[36], [37], [38], [39], [40]].

NGS is widely used in global and regional AMR surveillance [29,30]. Simple prediction of AMR involves classification of microbial strains as susceptible or resistant while added complex modelling approaches also aim to determine the MIC of an antimicrobial drug for a particular strain at which resistance is achieved. NGS coupled with machine learning greatly facilitates identification as well as prediction of AMR exclusively from genomic data without validation from tedious culture-based MIC assays [41,42]. Prevalent studies show that genomic data can predict AMR with sensitivity (determined by true positive rate) more than 87 % and specificity of more than 98 % (determined by true negative rate). AMR prediction through NGS involves identification of potential resistance-associated genes and cross-referencing them with databases of know AMR genes (rule-based approach) [31]. This can also enable identification of novel genetic determinants of AMR through correlation and network analysis. Detailed next generation microbial sequencing techniques is described in Fig. 4.

#### 1.5.1 Prediction tools & databases

Various pipelines have been designed to enable identification and prediction of pathogen-associated virulence factors and AMR using NGS data. It has been found that evolution of pathogen virulence and AMR, although distinct, share common characteristics. Both pathways equip microbes to adapt and survive in hostile conditions and it is commonly observed that both virulence and resistance determinants are co-transferred during HGT. Commonly used prediction tools and databases for identification of AMR genes and/or virulence factors include DeepARG [43], RGI [44], ResFinder [45], ARGsOAP [46,47], MP3 [48], VirulentPred [49,50], and PathoFact [51]. These tools differ in their capacity to predict AMR genes and/or virulence factors associated with the intrinsic genome, phage or MGEs. Prediction of the evolving AMR and/or virulence profile is critical to evaluate the pathogenic potential of a microbe. Studies by Kuang et al. [52] and Green et al. [53] adopted a combinatorial strategy of traditional machine learning approaches like logistic regression and random forests, along with convoluted neural networks which made significant improvements in drug resistance prediction and classification. The latter part of the review will delve into the specifics of machine learning approaches employed for predicting drug resistance in detail.

## 2 Application of NGS in AMR surveillance

Adopting an integrated strategy of genomic epidemiology, would allow examination of functional association between pathogen evolution and population dynamics which can delineate a trajectory for AMR evolution **(Box 6)**. NGS analyses of opportunistic human pathogen, *Staphylococcus aureus* and its associated methicillin-resistant strains (MRSA), has revealed a detailed map of how AMR strains can emerge and be maintained in populations at different spatiotemporal scales [54,55]. NGS has enabled construction of phylogenetic trees which revealed that MRSA emerged as early as 1940 through horizontal gene transfer of the staphylococcal cassette chromosome mec (SCCmec) element. This advent of AMR was attributed to unregulated use of penicillin at that time. Recent data has mapped out independent and parallel evolution of MRSA strains at different rates depending on their geographical distribution, but being largely common to hospital-related infections [55,56].

Emergence and spread of AMR is determined both by the rate of acquisition of resistance-associated mutations in the bacterial population and the fitness cost induced. NGS study of *Mycobacterium tuberculosis* showed that drug exposure resulted in acquisition of a number of resistance-associated mutations but these mutations were rarely transmitted because of compromised pathogen fitness. If a resistance-associated mutation exists with sufficient fitness, this mutation will be clonally transmitted throughout the population leading to emergence of a resistant strain [57,58]. Analyses have emphasized that geographical dissemination of AMR can be identified with AMR markers like SCCmec in MRSA. Tracing such AMR elements can help identify which resistant pathogens clones can persist regionally or globally. For e.g., CTX-*M*-15 is associated with the prevalence of resistant *Escherichia coli* ST131 [59] and several *Klebsiella pneumoniae* clones (CG14/15, ST101) [60,61].

Hospital-acquired infections account for majority of global infectious disease burden. Overwhelming microbial exposure with considerably attenuated immune defense against pathogens, create an environment that is amenable for development of antibiotic resistance [62]. NGS analysis have revealed several regional surges of AMR in the hospital patients infected with certain pathogens and in response to specific antibiotic regimes [[63], [64], [65]]. Accumulation of multiple AMR mutations resulting in multi-drug resistance is already established in MRSA and *M. tuberculosis* (super-bugs). Patterns of mutational events was largely overlapping in different patients in hospital acquired infections. This strongly suggests that AMR events are highly predictable. Examples include the repeated acquisition of SCCmec (methicillin resistance) [66,67], walKR mutations (vancomycin resistance) [68,69] in *S. aureus*, and lpx disruptions (colistin resistance) in *Acinetobacter baumannii* [70]. Multi-drug resistance to *Pseudomonas aeruginosa* constitutes one of the leading causes of mortality due to opportunistic infections. Hence, monitoring hospital outbreaks of *Pseudomonas aeruginosa* is crucial to characterize and combat evolution of AMR in these strains. Ramanathan et al. [71] performed NGS analyses of 10 multi-drug resistant clinical isolate of *P. aeruginosa* and identified novel non-polymorphic single nucleotide polymorphisms (nsSNP) associated with each isolate. These nsSNPs were enriched in various AMR genes including mexXY-oprM and mexAB-oprM genes coding for drug efflux pumps, conferring resistance to β—lactams, aminoglycosides and fluoroquinolones; in aminoglycoside modifying enzymes (AME's) and 16S rRNA methylase genes conferring resistance to colistin and β-lactams by modifying the bacterial outer wall. Further experimental validation of these SNPs in AMR by mutagenesis and proteomics evaluation and culture-based AST can help define *P. aeruginosa* strains at risk of developing AMR.

Use of NGS technologies for accurate identification of antimicrobial resistance phenotypes was further confirmed in a study involving WGS of more than 100 *Campylobacter* species isolates from human, meat and cecal samples of livestock animals [72]. The National Antimicrobial Resistance Monitoring System in the United States regularly monitors AMR in Campylobacter strains since they are major food-borne pathogens. WGS identified a total of 18 genes associated with AMR. Culture-based antibiotic susceptibility testing against a range of antibiotics confirmed that NGS-determined resistance genotype strongly correlated with culture-based resistance phenotype with correlations ranging from 95 % to 99 % depending on the antibiotic tested [72]. Hence, NGS-based genotyping can be used as powerful tool to identify and characterize resistance profiles and monitor AMR.

Recent use of an integrated approach of WGS and whole genome shotgun metagenomic sequencing allowed detailed characterization of a hospital outbreak of Carbapenem-resistant Enterobacteriaceae (CRE) [73]. Real-time PCR and WGS (Illumina sequencing) of bacterial isolates from burn patients confirmed the *bla*_IMP-4_ status of resistant *E. hormaechei* as origin of outbreak. Long-read SMRT sequencing (Pacific Biosciences) identified the genetic context of the *bla*_IMP-4_ on IncHI2 plasmid, commonly associated with this IMP-4-associated resistance across hospitals. The study traced this isolate to another previous outbreak in the same ward, strongly suggesting the hospital as the harboring source. This was confirmed by shotgun metagenomic sequencing of samples from the hospital environment. Similar integrated approach was also used to assess carbapenem-resistant *Acinetobacter baumannii* (CR-Ab) outbreak in a Brisbane hospital between 2016 and 2018 [74].

### 2.1 Application of NGS in AMR surveillance in infectious diseases

High-throughput and large-scale genomic screening has greatly expedited the process of identification of resistant and susceptible clinical pathogen strains, as well as detecting genetic elements conferring the resistance or susceptible property.

As discussed earlier, treatment of TB is significantly hampered by acquisition of multi-drug resistance. Hence, understanding mechanisms that underlie resistance is important for development of rapid tests to measure resistance, formulation of new drug regimens and re-purposing of existing drugs that will achieve better treatment outcomes. To this end, the Comprehensive Resistance Prediction for Tuberculosis: An International Consortium (CRyPTIC) performed GWAS (Genome wide association study) on 10,228 individual clinical isolates of *Mycobacterium tuberculosis* (Mtb) from 27 countries [75]. By correlating genotypic profiles with corresponding resistance traits, GWAS enables identification of resistance-associated genetic markers (both coding and non-coding), and is thus, useful for prediction of AMR. Whole genome sequencing (WBS) was performed along with semiquantitative determination of minimum inhibitory concentration (MIC) to 13 drugs that are used as first line or second line of TB treatment [75]. They managed to identify several unreported genomic variants at Rv1218c promoter binding site of the transcriptional repressor Rv1219c (isoniazid resistance); linezolid resistance upstream of the vapBC20 operon that cleaves 23S rRNA; clofazimine resistance in the region encoding α-helix lining the active site of Cyp142. Many newly identified variants associated with AMR were detected as rare variants at a minor allelic frequency (MAF) of less than 1 %. Hence, WGS can be adopted as a sensitive detection method for unexplored mechanisms of AMR. Use of GWAS analysis to identify potential markers of Mtb resistance was initially performed by WGS of 1452 clinical Mtb siolates along with MIC assay for 12 *anti*-TB drugs [76]. They found associations between phenotypic drug resistance and mutations both in protein coding and regulatory (non-coding) regions. They found previously unidentified 13 genetic loci associated with resistance phenotype which could be used as potential markers for AMR susceptibility. Another study used 2237 clinical isolates of Mtb which were categorized as resistant or susceptible based on computational predictions and phenotypes already reported [77]. Identified SNP and small InDel variants with a minor allelic frequency (MAF) > 1 % were subjected to GWAS analysis which identified upregulation of mutated genes involved in pathogen DNA repair pathways – MutY and UvB under antibiotic stress [77]. Most studies surrounding Mtb evolution and AMR resistance, use H37Rv reference strain to identify genomic variants and AMR mutational events [78]. Adopting a reference-agnostic computational modelling integrated with genetic interaction and protein structure analysis allowed detection of novel AMR determinants in 1595 Mtb strains [79]. This approach enabled detection of 24 unreported AMR gene targets and discovery of 74 epistatic gene interactions supported by structural information of affected proteins. This study found that mutations conferring potent resistance are concentrated in off-target genes like those which encode for transmembrane proteins. Also, there is high distribution of mutant AMR genes in regions where Mtb is widespread suggesting prolonged use of antibiotics. Interestingly, genetic makeup of the pathogen is not the primary determinant of AMR variation [79].

Similar GWAS approach identified genetic loci associated with penicillin and tetracycline susceptibility in clinical isolates of *Neisseria gonorrhoeae* which predominantly show lack of antimicrobial drug resistance [80]. Identification of antibiotic susceptible genetic determinants can provide fresh insight into the prevention of AMR dissemination as well formulating targeted drug routines. The study found presence of penA_01 chromosomal loci and absence of plasmid loci *bla*_*TEM*_ correlated with penicillin susceptibility. Presence of wild-type rpsJ codon 57 in combination with absence of plasmid loci *tetM* associated with tetracycline susceptibility.

## 3 Application of NGS in AMR surveillance in commensal microbes

The ecological and public health impacts of antibiotic resistance in commensal microbes are viewed in two contrasting ways. On one hand, commensal microbes are recognized as a significant long-term reservoir of genes and mutations responsible for drug resistance, which can be horizontally transferred to infecting pathogens, thus substantially contributing to the expansion and dissemination of AMR [81,82]. Metagenomic sequencing of 162 human gut samples of Chinese, Danish and Spanish individuals revealed differential abundance of resistance genes across nations and presence of >1000 unique resistance genes predominantly observed in *Firmicutes*, *Bacteroidetes* and *Proteobacteria* populations of the gut [83]. Patterns of resistance gene enrichment were largely non-overlapping across geographical regions. WGS and microarray analysis of the oral microbiome of healthy individuals also showed high prevalence of resistance against macrolides, tetracyclines, streptogramin and lincosamides [84]. Resistance to macrolides, beta-lactams and tetracyclines is commonly observed in healthy commensal microbial communities.

Additionally, under non-homeostatic conditions such as chronic inflammation and immunodeficiency, commensal microbes can cross tissue barriers and invade the bloodstream, causing significant tissue damage, thereby acting as conditional or opportunistic pathogens. High-throughput chromatin capture (Hi-C) metagenomic sequencing of gut microbiomes of healthy individuals and transplant patients undergoing prolonged antibiotic therapy, has uncovered distinct networks of horizontal gene transfer of drug resistance genes and mobile genetic elements across individuals [85]. In healthy individuals, baseline gene transfer was prominently observed within gut microbial communities of the same phylum. In contrast, neutropenic transplant patients displayed an emergence of novel resistance genes and increased gene transfer both within and between different phyla. Notably, gut-resident Enterobacteriaceae exchanged genes with opportunistic pathogens like *Veillonella parvula* and *Enterococcus faecium*. Furthermore, a denser gene transfer network was associated with lower microbiome diversity, as observed during antibiotic treatments, promoting gene exchange among closely related community members. Exchange of gene cassettes coding for multidrug resistance against non-administered beta-lactams and fluoroquinolones was observed thus, resulting in significant expansion of the host commensal ‘resistome’ [85]. In another study by Förster et al. [86], pairwise gene comparison was performed between >1300 commensal genomes obtained from the Human Gastrointestinal Microbiota Genome Collection (HGG) and >45,000 publicly available genomes of pathogenic strains. Resistance genes acquired through HGT event would show significant homology of their sequences, regardless of the phylogenetic relationships of the participating strains. >64,000 gene transfer events, shared across commensals and pathogenic strains were identified as antibiotic resistance genes (ARGs). These ARGs were predominantly associated with resistance to beta-lactamases, aminoglycosides, antimicrobial peptides and multidrug efflux proteins. A small but significant proportion (1.5 %) of horizontally acquired resistance genes showed exchange events across multiple phyla and hosts, thus providing strong evidence of widespread mobilization of AMR from the commensal reservoir. Microbiome sampling from human nasal cavity, vagina and skin, along with ruminant gut revealed similar HGT of resistance genes, thus calling for close surveillance of diverse commensal niches for AMR dissemination [86].

On the other hand, numerous studies have shown that commensal microbes can prevent pathogenic infections by promoting colonization resistance [87]. They achieve this through competition and active receptor or metabolite signaling, which inhibit the entry, survival and evolution of drug-resistant pathogens. Indeed, it has been demonstrated that antibiotic treatments disrupt host microbiome (dysbiosis), which facilitates outgrowth of antibiotic-resistant target pathogens (competitive release) or, more commonly, bystander pathogens residing in individuals with chronic infections [88]. Accordingly, a study by Annavajhala et al. [89], employed 16S rRNA metagenomic sequencing of fecal samples of liver transplant patients at pre- and post-transplant time-points. Dramatic reduction in the alpha diversity of microbial communities along with significant alterations of the community composition was observed post-antibiotic treatment. Significant associations were found between altered microbiome diversity prior to transplant and subsequent colonization of enteric multidrug resistant bacteria (MDRB), predominantly consisting of third-generation cephalosporine- and carbapenem-resistant Enterobacteriaceae along with vancomycin-resistant Enterococci. Moreover, pre- and post-transplant temporal patterns of drug-resistant enteric pathogen colonization was correlated with the class of antibiotic used. This can be used to predict evolution of microbiome diversity, selection of resistance markers and associated long-term clinical complications.

Administration of antibiotics is also associated with widespread *Clostridium difficile* infection (CDI) [90]. Its treatment results in emergence of MDR isolates of this pathogen that are associated with significant morality. Studies using metagenomic analysis of human fecal specimens have shown successful mitigation of refractory CDI through restoration of the commensal microbiome by fecal microbial transplantation (FMT) [91,92]. The microbiome of patients with recurring CDI were predominated by pathogenic *Proteobacteria* species. FMT resulted in expansion of the commensal *Bacteroidetes* and *Firmicutes* and mitigation of drug resistant infections [91]. A long-term study of stool samples from MDR-TB patients undergoing broad-spectrum treatment revealed concurrent evolution of drug resistance in both pathobionts and commensals [93]. Expectedly, there was a depletion of beneficial commensal communities such as *Bacteroides*, *Blautia*, *Clostridium*, *Eubacteria*, and Faecalibacterium, along with a simultaneous expansion of pathogenic *Klebsiella*, *Citrobacter*, *Enterococcus*, and *Escherichia coli* during treatment. Although there was a notable decrease in microbiome diversity lasting up to six months, diversity was largely replenished following the completion of treatment. Subsequent genome analysis, including SNP detection, showed that the restored microbial communities consisted of strains different from those present before treatment. There was high prevalence of genes coding for drug resistance in both pathogens and commensals. However, the superior fitness and resilience of the resistant commensal microbiome community enabled them to outcompete the resistant pathobionts, leading to the resolution of TB. Thus, this study offers an interesting and unexpected insight that antibiotic resistant commensal strains are beneficial for elimination of resistant pathogenic strains. The diverse impacts of the microbiome on either reducing or amplifying the host resistome are influenced by various factors. These encompass individual-specific antibiotic treatments, the composition of the microbiome before and after treatment, which affects the colonization potential within the microbiome niche, and the choice of sampling source (whether gastrointestinal mucosa or stool samples. Additionally, it is essential to thoroughly analyze these parameters to assess the microbiome's role in the dissemination of AMR [94].

## 4 Clonal expansion and global dissemination of MDR pathogens

Many MDR pathogens exhibit community transmissions with a strong trend towards emergence and dissemination of clonal lineages [95,96]. Local clonal expansion of MDR pathogens is evident in nosocomial infections, driven by higher frequency of transmission and exposure to common sources of infection in confined spaces or closed communities. A study by Murase et al. [97], uncovered clonal lineages of MDR and extensively drug-resistant (XDR) *Mycobacterium tuberculosis* strains from patient samples in Japan in 2002. 38 % of the strains could be classified into 9 clusters that were characteristic of local geographical identity. The higher clonality of XDR strains in comparison to non-XDR strains strongly suggested community transmission and clonal evolution of AMR. Epidemic clonal lineages or international clones, on the other hand, are genetically distinct populations of a pathogen species that are spatially segregated, indicating their successful transmission outside local communities and across geographical regions. One notable example is the evolution of multidrug-resistant *Acinetobacter baumannii* which accounts for approximately 2 % of hospital-related infections in U.S.A. and Europe and 4 % in Asia. Its evolution is mainly driven by globally disseminated international clones, mainly IC1, IC2, IC6 and IC9, accounting for approximately 77 % of carbapenem resistance [98]. Similarly, high-risk international ESBL-producing *E. coli* clones consisting of varying frequencies of ST405, ST468, ST410, ST38, ST73 and ST1193 have been observed in circulating *E. coli* populations in Bangladesh, in addition to the highly pandemic ST131 lineage [99]. These isolates were involved in extraintestinal infections and strongly associated with hospital-related infections. The predominant prevalence of international clones/STs of hospital-associated methicillin-resistant *Staphylococcus aureus* (HA-MRSA), extended-spectrum β-lactamase-(ESBL)-producing *Klebsiella pneumoniae*, and *Clostridioides difficile* can be attributed their ability to acquire and tolerate huge burdens of resistance-conferring mutations without incurring any appreciable fitness cost [100]. The two most widely prevalent HA-MRSA international lineages include ST22 and ST8 which are characterized by double-serine mutations at Ser84 in gyrA (coding for DNA gyrase) and Ser80 in grlA genes (coding for DNA topoisomerase IV) [101,102]. Both these mutations confer high resistance to quinolones and strains harboring them show high fitness. In contrast, minor clones experience significant fitness disadvantage upon acquisition of resistance-causing mutations, resulting in their impeded global dissemination. Double-serine mutation in Ser83 if gyrA and ser80 of parC is also observed in international clonal lineages of ESBL-producing *K. pneumoniae*, a feature that was distinctly lacking in minor lineages [102]. Similar emergence of major international resistant clones of Mtb have also been examined. Loiseau and colleagues [103] demonstrated that specific lineages of *Mycobacterium tuberculosis* exhibit varying capabilities of transmission, with some lineages being more proficient in widespread dissemination across nations, while others remaining more inclined towards local emergence and persistence. Their research revealed that the MDR Beijing/L2 clones circulating in Georgia displayed heightened transmission rates. Surprisingly, MDR L4 clones exhibited slower transmission rates even when compared to their drug-susceptible counterparts. The enhanced transmission rates were attributed to epistatic interactions involving the rifampicin resistance-conferring mutation RpoB S450L, compensatory mutations in the RNA polymerase, and other inherent genetic traits of the L2/Beijing clone, which collectively conferred increased adaptive fitness to these clones.

High abundance of international AMR clones renders the prevailing notion of regional endemic AMR clones obsolete and strongly suggests redirection of efforts towards surveillance of global epidemic and pandemic clones under the purview of One Health framework. Emergence of MDR pathogens is strongly correlated with use of specific classes of antimicrobials like fluoroquinolones. Although limiting the use of antimicrobials may not entirely eliminate the emergence of AMR, it has the potential to restrict the spread of major international resistant clones. Therefore, the global monitoring with emphasis on these predominant lineages should be incorporated into the One Health strategy to develop measures aimed at curbing their widespread geographical dissemination.

### 4.1 Application of NGS in AMR surveillance of environmental and ecological niches

**Fig. 5:**
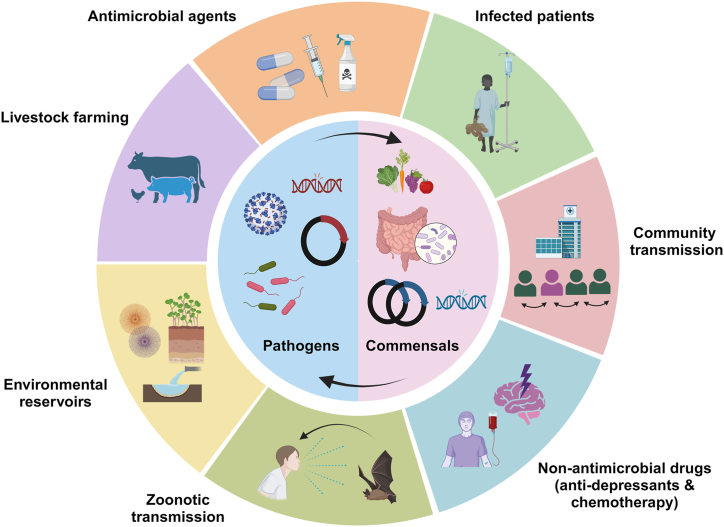
**Determinants of antimicrobial resistance.** Global prevalence of AMR in microbes is a complex interplay of microbial-intrinsic, host-intrinsic and environmental factors. Despite AMR being a natural occurrence, unimpeded drug consumption has exerted enhanced evolutionary pressure resulting in overwhelming growth of AMR in microbes. Due to ease of acquisition and transfer of drug resistance elements in microbial communities, it is essential to monitor not only microbial communities in human samples, but also in environmental sources like sewage, water, fecal matter, livestock and air pollutants. Hence, genomic surveillance of all these microbial AMR sources is necessary for prediction of global AMR trend.

Various host-intrinsic and extrinsic/environmental parameters, which include biotic and abiotic components, should be taken into account during prediction of regional and widespread AMR trends (Fig. 5).

#### 4.1.1 Sewage sampling

Current approaches for AMR surveillance are based on data from spatially confined sources like hospitals or community infections, which may not provide a comprehensive representation of the entire network of genes associated with resistance. Study by Hendrikson et al. [104] included metagenomic analysis in untreated urban sewage samples from 79 sites of 60 countries. The microbial resistome of these areas, showed dynamic changes in abundance and diversity depending on geographical location, socioeconomic, health or sanitation and environmental parameters. Genomic analysis involved estimating global distribution of AMR genes, diversity and clustering of those genes and associated drug classes. Surprisingly, patterns of antimicrobial use only had a minor contribution to the development of associated AMR in this study. Rather, the Human Development Index (HDI) was found to be a stronger predictor of population-based AMR.

Another study by Munk et al. [105], involved metagenomic analyses of 757 urban sewage samples distributed across 101 countries. As expected, abundance of antimicrobial genes (ARG) showed geographical skewing, with greatest abundance in the sub-Saharan Africa. While seasonality had little impact on resistome profiles, higher bacterial diversity correlated with higher ARG diversity. Drug class usage and associated resistance also showed substantial geographical variations. *Proteobacteria* and *Firmicutes, Bacteroidetes* and *Actinobacteria* almost entirely accounted for the bacterial ARGs. Evaluation of phylogenetic relationships between ARGs and the flanking genes (taxonomic scaffolds) across samples can provide a crucial insight into the evolutionary landscape of sample resistomes. A stable resistome can be observed as ARGs flanked by similar sequences across samples. Mobile or divergent ARGs on the other hand will be found across samples, but in different genomic contexts. Of the 49 ARGs analyzed, different ARGs showed varying levels of stability or divergence across samples. AMR prediction on the basis of taxonomic scaffolds of ARGs is limiting in plasmid-associated resistance genes [105]. Hence, different strategies to predict dispersal and evolution of resistance due to HGT needs to be formulated [106].

Urban sewage sampling is increasingly gaining traction as it can greatly augment AMR prediction by potentially identifying alternate reservoirs of ARGs that are overlooked during sampling of hospital-admitted patients.

#### 4.1.2 Air sampling

A recent insightful study by Zhou et al. [107] found significant positive correlation between levels of air particulate matter PM_2.5_ and antimicrobial resistance associated with 11.5 million isolates of nine pathogens. This relationship also showed temporal significances. PM_2.5_ can be disseminated through wider regions and can be easily inhaled. There is also higher abundance of PM_2.5_ in environmental sources including, soil, river and sewage treatment plants. Because of their widespread abundance, they actively mediate HGT and spread of resistance. Hence, global air monitoring should also be considered during AMR prediction.

#### 4.1.3 Livestock and food sampling

The World Health Organization has listed 12 species of bacteria as global priority pathogens (GPP) classified as critical, high and medium priority with regards to their antimicrobial resistance profiles (World Health Organization. 2017). AMR surveillance shows predominance of data from human sources. Food and livestock form two equally major aspects of global AMR trend. Zoonotic infections comprise about 60 % of known infectious diseases and up to 75 % of new infectious diseases. Reverse zoonoses account for more than 50 % of human pathogens [108]. A systems approach, ‘ONE Health’ is being increasingly adopted to integrate AMR data from environmental, zoonotic and human sources to combat global AMR [109]. Veterinary antibiotic consumption is estimated to be 1000-fold higher when compared to human consumption. Dysregulated antibiotic use for livestock production is a major threat to global dissemination of AMR [110]. Antimicrobials administered to animals manage to make way to their surroundings through excreta which results in alteration of resistance profiles of the environmental microbiome [111]. Entry of these altered microbes into the food chain leads to widespread propagation of associated resistance profiles to human population [112,113]. AMR spread to the food chain occurs directly through consumption of contaminated animal products. For e.g., AMR associated with *Salmonella typhimurium* infections has been traced to consumption of contaminated meat and poultry [114,115]. Similar findings were observed for AMR in *Campylobacter* and Quinolone-resistant *E. coli* [116]. AMR-associated with non-animal food consumption has also been associated with outbreaks between 2007 and 2011 and could be attributed to consumption of contaminated food due to dissemination of resistant pathogens [116]. Moreover, many food processing and preservation techniques require use of microbes which are not screened for their resistance profiles and thus, can make way into the food chain. Many food-processing techniques also require killing of microbes whose DNA can be taken up by other microbes resulting in transfer of resistance elements.

A study involving genome sequencing of 168 isolates, primarily *Escherichia coli*, from livestock fecal matter in 14 farms of United Kingdom explored the relationship between AMR burden and antimicrobial drug use in the farms [117]. They found that while use of increasingly diverse classes of antimicrobial drugs led to development of MDR strains, host-related factors also determined AMR burden and transmission in these populations.

A study by Zheng et al. [118] revealed that ARG abundance was higher in soil samples from agricultural habitats than non-agricultural habitats. Both pathogenic and host-commensal microbes facilitated dissemination of ARGs. Hence, monitoring agricultural practices across different geographical regions including patterns of use of antibiotics, pesticides, treatment of wastewater irrigation and livestock is paramount to combating this source of global antimicrobial resistance.

#### 4.1.4 Gut microbiome

Human and animal gut microbiota form a significant and stable source of antimicrobial resistance through widespread HGT between bacterial pathogens and host commensals [81,119]. Hence, monitoring of dietary intake and associated gut microbiome is crucial in the AMR prediction pipeline [120]. Current metagenomic approaches have facilitated identification of the host microbiome profiles. But prediction of novel ARGs, especially ones associated with microbial host-specific mobile genetic elements is challenging with current metagenomic tools. Yaffe and Relman [121] utilized metagenomic high-throughput chromosomal confirmation capture (Hi-C) to evaluate multiple microbial genomes in the gut of 218 adults from samples collected 10 years apart. From the metagenome-assembled genomes (MAGs), core and accessory genes were characterized with core genes bearing similarity to more than 90 % of the reference genome. Widespread in-situ exchange of accessory elements was found among microbial communities as a result of HGT, as well as significant adaptive evolution under selection pressure over 10 years in the core genome. In fact, Hi-C in human gut microbiota identified distinct networks of HGT of AR genes and mobile genetic elements that were associated with spread of AMR [85]. The transfer networks varied across individuals. While healthy individuals showed background level of genetic exchange; inflammation and neutropenia resulted in higher pathogen abundance and consequently denser HGT networks associated with diverse microbial community [85]. So, examination of the host immune response to limit pathogen load, can provide critical insights into control of AMR evolution within the host commensal-pathogen community.

### 4.2 Prediction of AMR using phylodynamic-based modelling

Mere NGS-enabled identification of acquired resistance-conferring mutational events and mobile genetic elements in microbial populations remain largely inadequate for AMR surveillance. AMR dissemination involves a complex interplay of interconnected events, including the genetic evolution of microbes, their survival and proliferation under intense selection pressures (such as host immunity), and the successful transmission of resistance genes between microbial communities and hosts populations. Tracking microbial evolutionary pathways and transmission rates within and between microbial and host populations require a comprehensive approach involving merging evolutionary biology and epidemiology, known as “phylodynamics” [122]. This approach was initially developed for monitoring viral infections and prediction of regional or global outbreaks. Its successful implementation during global tracking of emergence of SARS-CoV-2 genetic variants and their transmission dynamics, especially those of variants of concerns (VOC) [123] guided public health strategies.

Phylodynamics is based on the implicit assumption that molecular evolution of a pathogen occurs on the same timescale as its transmission [122,124]. Therefore, the observed branching pattern of its phylogenetic tree will closely correspond to its transmission network pattern, thereby reflecting its transmission dynamics. Each branch length of a phylogenetic tree can, therefore be converted to time using the evolutionary clock rate as the conversion factor [124]. This enables correlation between epidemiological and evolutionary rates in phylodynamics modelling and thus, prediction of transmission dynamics through genomic evaluation of global microbial populations.

**Fig. 6:**
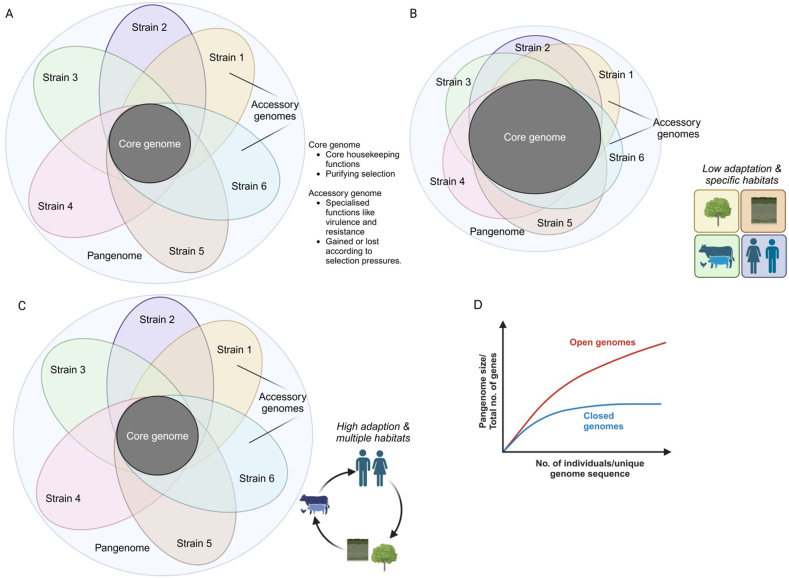
**Dynamics of bacterial pangenome architecture. A**. The highly complex bacterial genome is evident by its high intra-species genetic diversity. And comprises a core of set genes that are common to every member of a specific species. They coding for essential housekeeping functions like transcription and translation. The accessory genome is genetic component that is unique each member of a bacterial population. This part of the genome remains dispensable and subject to acquisition or loss under selective pressures. The pangenome consists of the complete repertoire of genes found within all the members of the bacterial population. **B.** Some bacterial species are characterized by a large core genome and small accessory genome resulting in overall small pangenome size with limited genetic variation its among members. These species, known as ‘specialists’ have closed pangenomes and show limited adaption to varying ecological niches. **C.** Many bacterial species display smaller overlapping core genome along with expansive accessory genome. These ‘generalist’ species possessing open pangenomes, exhibit considerable genetic heterogeneity among its members and a superior ability of genetic adaptation to persist in ecologically distinct niche. **D.** Open pangenomes tend show exponential increase in size with addition of new genetic entities (strains). While closed pangenomes contains a finite number of genetic sequences and addition of new members do not result in any appreciable increase in the overall pangenome size or genetic variability.

Phylodynamics-based modelling is highly applicable to RNA viruses because of its high mutation frequency, large population size and high replication rate or generation time which ensures similar timescales of genetic evolution and epidemiological processes [122,125]. The shorter timescales of RNA virus phylogenetic evolution ensure that its genetic sampling at any timepoint will provide rich information characterizing new transmissions and outbreaks. It is, however, challenging for monitoring and predicting bacterial AMR as epidemiological processes that drive bacterial spatiotemporal patterns and phylogenetic processes that govern rates of individual population evolution operate on vastly different timescales [125]. Moreover, bacterial populations exhibit a highly complex pattern of genomic evolution compared to viruses. Bacterial genome structure is dynamic and consists of a core, accessory and the pangenome (Fig. 6A) [126,127]. The ‘core genome’ codes for essential functions and is the conserved genetic component common to all bacterial isolates of a species. The ‘accessory genome’ of an isolate can be modified, gained or lost depending on selection pressures. The entire complement of genes within a bacterial species is referred to as the ‘pangenome,’ which encompasses a repertoire significantly larger than that found in any individual bacterial genome. Bacterial populations can be generally categorized under two categories: specialist and generalists [125]. ‘Specialists’ have highly closed or finite pangenomes and are characteristic of bacteria that have strictly adapted to their inhospitable ecological niche (Fig. 6B). Generalists, on the other hand, have open and larger pangenome that enables them to thrive in a range of hosts and ecological niche (Fig. 6C). The pangenome's size of a social specialist varies according to the local diversity of its habitat, where higher local diversity favors sizes larger than that of a social generalist [128,129]. Hence, survival and persistence of specialists versus generalists are intricately linked to diversity of the local niche, allowing their differential kinetics of evolution and spread. High frequency of occurrences of ‘generalists’ or ‘specialists’ in niche-specific reservoirs for extended time periods might result in their sporadic outbreaks, which can hinder accurate estimation and prediction of their transmission dynamics. Open pangenomes exhibit dynamism by continuously expanding in size with the incorporation of new genomic entities. Consequently, endeavors aimed at sequencing additional strains within the pangenome are more likely to unveil novel genes, a scenario less probable in closed pangenomes (Fig. 6D). AMR surveillance focuses on understanding mechanisms of evolution of core genomes and needs to incorporate the evolving accessory genome and pangenome data. Phylodynamics-based predictions also assume that transmission occurs between host populations at random making every host equally susceptible to infection [125]. Contrary to this assumption, AMR transmission is not entirely stochastic and depends on a number of host-intrinsic (such as host immunity) and external socioeconomic factors; rendering certain populations segregated in time and space, more susceptible.

There is a large reserve of research dedicated to formulating and improving current computational platforms of phylodynamics-based predictions of pathogen evolution and spread reviewed extensively [124,130].

### 4.3 Challenges in phylodynamics-based AMR prediction

There are a number of variables that pose significant challenges to phylodynamics-based modelling of pathogen evolution and transmission which are applicable to AMR prediction strategies. They include spatial and temporal sampling biases which can negatively impact inferences of transmission dynamics or ‘phylogeography’ of a pathogen [131,132]. Phylodynamics assume lack of any genetic recombination within hosts population which would potentially undermine the impact of microbes that had distinct time and geographical origins of emergence, coexisting within a single host at any time-point [124,133,134]. This would have significant consequences in elucidating and predicting the AMR profiles of host populations. Phylodynamics-based modelling is concentrated on pathogen genotypes. But phenotypic characterization of its host specificity, antigenicity affecting host immunity and intra-host replication rate must also be incorporated to determine its transmission dynamics [131,135]. Moreover, the microbial diversity and their evolutionary heterogeneity within host might result in discordant patterns of intra-host (faster) and inter-host AMR (slower) evolution [125,136].

AMR profiles of ‘specialists’ versus ‘generalists’ shown distinct patterns. Specialists like *Neisseria gonorrhoeae* and *Mycobacterium tuberculosis* [137] commonly display an enrichment of point mutations driving MDR phenotypes. In contrast, generalists like *E. coli* harbor MGE like plasmids and transposons that encode multiple resistance genes. MGE's higher propensity for HGT and lower incurred fitness costs enables them to survive and transmit across a greater diversity of hosts species [125,138]. Sequencing of context DNA flanking the mcr-1 gene that confers colistin resistance traced the origin of its current widespread global distribution. Mcr-1 gene showed strong association with ISApl1 transposon whose initial mobilization occurred in 2006, followed by subsequent population expansion and detection of mcr-1 resistance in 2011 [139]. Hence, prevalence of distinct mechanisms of genetic modifications in distinct geographical niche can potentially influence evolution and dissemination of AMR, and should be incorporated as important variables in current phylodynamics-based prediction models. Hyun et al. [140], recently evaluated the pangenome of 12 bacterial species, aiming to elucidate the interrelationships governing the evolution of antimicrobial resistance (AMR) among them. This comprehensive analysis encompassed a vast pangenome of over 27,000 genomes from the 12 species, which were evaluated against a panel of 69 drugs. The findings of the study revealed frequent mobilization of AMR genes between closely related species with marked reduction in transmission between phylogenetic classes. Their study successfully implemented machine learning at predicting AMR phenotypes with higher accuracy and sensitivity than prevailing GWAS-based detection. While inclusion of the entire pangenome data in these models might make it computationally intensive, one could make use of targeted regions within the pangenome, such as those associated with virulence and resistance in phylodynamics-based AMR prediction.

### 4.4 Application of artificial intelligence (AI) techniques in AMR prediction

AMR detection and prediction using genotypic data can be accomplished through rule-based methods or machine learning (ML) and deep learning (DL) techniques [41]. The rule-based approach relies on pre-existing knowledge of how resistance develops in a particular microorganism, including the identification of known resistance genes and mutations. It is not well-suited for detecting new resistance factors or dealing with complex resistance mechanisms. Conversely, ML and DL approaches can effectively utilize large datasets of high-quality genomic data from diverse isolates to make accurate predictions, even when faced with novel or complex resistance mechanisms [141]. NGS applications leveraging artificial intelligence (AI) techniques, including ML and DL, are increasingly utilized for predicting and monitoring AMR. By analyzing extensive data repositories on antibiotic usage and corresponding resistance patterns, ML and DL algorithms can identify trends in emerging resistance and detect potential AMR hotspots. These advanced AI models enable predictions about which pathogens are likely to develop resistance to specific antibiotic classes. Furthermore, by examining the relationships between antibiotic consumption and resistance development in various clinical settings, AI can optimize antibiotic regimens, enhancing treatment efficacy in healthcare [142].

ML/DL methodologies entail the mapping of input data sourced from isolate-specific shotgun DNA, metagenomic DNA, or amplicon sequences to corresponding output data associated with phenotypic antibiotic susceptibility testing. Through this process, they aim to elucidate and capture the intricate non-linear relationships and patterns inherent in the data. Additionally, transcriptional response data induced by antibiotics can be integrated effectively, incorporating both phenotypic and genotypic information for more robust AMR prediction. Supervised learning paradigms involve the training of models on input features alongside their respective target outputs, facilitating the discovery and approximation of underlying non-linear relationships [143,144]. Such models are especially advantageous in tasks involving regression and prediction, rendering them well-suited for AMR monitoring endeavors. ML models utilized for the classification and prediction of AMR include a range of techniques, including logistic regression, support vector machines, random forests, decision trees, gradient boosting models, and neural networks [[145], [146], [147], [148]]. The dataset is typically divided into training and test sets, where the former is employed for model approximation, and the latter serves for evaluating model accuracy. Various metrics such as root mean square error and correlation function are examined for regression models, while classifier problems are assessed using metrics like confusion matrix, accuracy, recall, and sensitivity to evaluate model accuracy [143]. Detailed methodology of ML/DL approach of AMR prediction has been extensively reviewed elsewhere [41,141,149]. Significant advancements have been made in AI-driven antimicrobial resistance (AMR) prediction. Arango-Argoty et al. [42] developed two deep learning models, DeepARG-SS for short read sequences and DeepARG-LS for long read sequences, to identify and predict AMR genes from metagenomic data. These models employ neural networks trained on existing datasets of known antibiotic resistance genes (ARGs) to identify and potentially classify novel resistance genes. DeepARG has demonstrated high precision rates exceeding 0.97 and recall rates over 0.90, showcasing its effectiveness in accurately detecting resistance genes. While many machine learning (ML)-based investigations concentrate on predicting resistance to individual drugs, there's a notable absence of studies tracking MDR and the longitudinal progression of resistance traits. An impactful investigation by Ren et al. [150] introduced a multi-label classification (MLC) strategy to model multidrug resistance in pathogenic *E. coli* against ciprofloxacin (CIP), cefotaxime (CTX), ceftazidime (CTZ), and gentamicin (GEN). Employing a comparative approach, the ensemble classifier chain (ECC) model within the MLC framework demonstrated superior accuracy in identifying and predicting secondary chromosomal mutations associated with resistance. Remarkably, this model effectively operated without prior knowledge of specific resistance genes. Notably, the model's performance exhibited variation dependent on the drug, with the highest accuracy observed for CIP and the lowest for GEN. This divergence in performance could potentially be attributed to the genetic underpinnings of resistance, where resistance to GEN is predominantly driven by mobile genetic elements (MGEs), while the modelling efforts here primarily focused on chromosomal mutations.

ML/DL methodologies require a substantial volume of samples exhibiting a balanced distribution across resistance phenotypes (including susceptible, intermediate, and resistant) to establish a robust, precise, and broadly applicable model during the training process [141,149]. Nevertheless, datasets typically suffer from skewness, with a limited number of samples available for novel antibiotics or novel genes and mutations. To address these challenges inherent in conventional ML/DL approaches, Ren et al. [151] employed transfer learning methodology. Utilizing WGS datasets of human and animal *E. coli* isolates and resistance information for Ciprofloxacin (CIP), Cefotaxime (CTX), Ceftazidime (CTZ), and Gentamicin (GEN), a fundamental convolutional neural network (CNN) was implemented for each antibiotic. The CIP model, distinguished by its balanced nature and heightened accuracy, surpassed the others and thus served as the pre-trained model (source domain). This knowledge was subsequently transferred to enhance the AMR prediction capabilities of the remaining antibiotics (target domain). Transfer learning yielded significant improvements in AMR prediction accuracy, surpassing conventional CNN approaches. Similarly, Hyun et al. [152], designed a ML workflow for identifying genetic drivers of AMR by constructing a reference strain-agnostic pan-genomes of 288 *Staphylococcus aureus*, 456 *Pseudomonas aeruginosa*, and 1588 *Escherichia coli* isolates and training random subspace ensembles (RSEs). RSEs utilize a subset of features from the original sample dataset (genomes associated with ANR phenotypes) and feature set (genes and alleles within those genome) to train each base model. Predictions from each base model are eventually combined to improve overall performance and robustness of the final prediction. When employed to analyze 14 drugs, the RSE approach demonstrated superior performance compared to conventional statistical methods in detecting and identifying both existing and novel resistance genes.

Machine learning (ML) approaches can be effectively utilized for antimicrobial resistance (AMR) surveillance. Kaur et al. [153] used linear regression modelling to analyze nationwide AMR trends, considering time and hospital-specific variations. The study identified high resistance to Imipenem in *Acinetobacte*r, *Klebsiella*, and *E. coli* bloodstream infections (BSIs). Distinct regional trends of antibiotic resistance against various pathogens were also noted. A significant temporal link was found between community-acquired and hospital-acquired infections for *Klebsiella* and *Acinetobacter* species. Cefotaxime resistance emerged as a potential early warning sign for increasing carbapenem resistance in both hospital and community settings. Additionally, a data-driven Bayesian network ensemble, averaged over 301 bootstrap samples, was created to uncover systemic associations between AMR and Sustainable Development Goals (SDGs). The study found that states with higher achievement of SDG3 (good health and well-being) had lower Imipenem resistance in *Klebsiella* BSIs. This model-based AMR scorecard can be used to enhance and focus strategies of AMR surveillance. This data-driven AMR surveillance framework aligns with WHO GLASS standards, making it adaptable for other low- and middle-income countries and hospitals adhering to these guidelines.

AI-enabled AMR prediction encounters significant challenges. These encompass the availability of high-quality data and the necessity for balanced representations of susceptible, resistant, and intermediate phenotypes across a wide range of MICs. Prediction accuracy also varies depending on the type of data and modelling algorithm utilized. Sampling discrepancies due to geographical disparities and experimental conditions can further impact result reliability [141]. Additional research is required to address these concerns, paving the way for the implementation of modelling-based AMR prediction in clinical, diagnostic, and surveillance settings.

### 4.5 Challenges in genomic surveillance of AMR within the one health framework

Global genomic surveillance and prediction of AMR warrants uniformity in standardization of technologies and modelling-based predictions to ensure reproducible results across samples collected from heterogenous sources and geographically segregated habitats. However, significant technological and analytical limitations greatly hinder the ease and accuracy of AMR prediction on a global scale.

As previously described, metagenome sequencing remains in its nascent stages and faces challenges such as low coverage and high background noise [18]. Shorter reads in shotgun metagenomics hinder accurate genome resolution, especially in regions enriched with repeats [154]. While whole-genome sequencing (WGS) of culturable bacterial isolates addresses these limitations, it is costly and restricted to culturable bacterial strains. Advent of long read metagenomics platforms like Oxford Nanopore and Pacific Biosciences, along with advancements in genomic assemblers that produce high quality metagenome-assembled genomes (MAGs) have provided significant momentum in this direction [155,156]. These technologies, however, become inadequate at distinguishing high diversity strains due to mis-assembly of metagenomic contigs, and most MAGs fail to recover resistance-coding plasmids [145,154]. While long read and hybrid assemblies had no significant impact on the overall quality of genome assembly, hybrid assemblies were more efficient at assembling certain genomic regions like the 16S rRNA gene and also showed superior performance at distinguishing closely related strains in the pooled metagenome [157].Recent techniques like Hi-C [158] and detection of DNA modifications like methylation [159,160] have provided hope for strain-level classification and plasmid association. Delineation of the complete repertoire of microbial diversity and associate genetic resistance profiles will facilitate optimal modelling and prediction of evolutionary and transmission dynamics of AMR. The Initiative for the Critical Assessment of Metagenome Interpretation (CAM) is a community-driven endeavor aimed at unbiased evaluation of methods related to assembly, taxonomic profiling and binning [157,161]. The goal is to ensure high level of consensus and reproducibility in interpreting metagenomic data which will significantly enhance use of genomics in routine clinical practices. A recently discovered drawback of genetic-based AMR prediction is lack of concordance between genotypic and phenotypic AMR profiles in a reasonable percentage of microbes. Rebelo et al. [162] performed phenotypic AST on 500 random bacterial isolates from the Danish Clinical Laboratories and compared the results to resistance predictions generated from WGS and in-silico prediction using the ResFinder database. 91.7 % of all isolate-drug combinations showed concordance between phenotypic testing and genotypic prediction. 6.2 % corresponded to antibiotic-susceptible isolates harboring AMR genetic elements. The remaining 2.1 % of all isolate-antimicrobial combinations was formed of phenotypically resistant isolates that lacked any known AMR genetic determinants. This 2.1 % corresponded to a significant 26.4 % of all resistant isolates identified by phenotypic testing. Certain combinations of species-antimicrobials including macrolides and tetracycline in streptococci, ciprofloxacin and b-lactams in combination with b-lactamase inhibitors in *Enterobacterales*, and most antimicrobials in *Pseudomonas aeruginosa* were more susceptible to discordance between phenotypic testing and genotypic predictions [162]. Hence, clinical settings of AMR surveillance cannot completely exclude phenotypic testing of resistant profiles. Genotypic predictions cannot detect novel genetic determinants of resistance. Iterative kmer-based analysis and machine learning are being developed towards this end, but their output is heavily contingent upon the type of reference genomes used and require phenotypic testing for validation [28]. Large datasets of MIC distribution should be periodically compared with genomic sequencing. This is essential for constant updates in databases of resistance-conferring genes and plasmids, and exclusion of genetic determinants that do not confer any phenotypic resistance from current databases.

Genotypic prediction of AMR is heavily dependent on databases harboring information on genetic determinants of resistance. These databases, however, show significant differences in their architecture, genetic repertoire of resistance, annotation tools and associated metadata Papp and Solymosi [163]. Inclusion criteria for AMR genetic determinants (such as published in scientific literatures), type of genetic determinants (chromosomal mutations versus horizontally acquired genetic material), entry format (FASTA or FASTQ; nucleotide versus amino acid sequence; raw reads versus assembled reads through different assemblers) and accessibility vary across databases and potentially results in variable outputs of predicted AMR profiles. Papp and Solymosi [163] have extensively reviewed advantages and limitations of frequently used AMR databases. According to their assessment, National Database of Antibiotic Resistant Organisms (NADARO) contains the highest number of horizontally acquired resistance genes which can be potentially superior at evaluating mobilization of resistance genes during environmental surveillance of AMR. Comprehensive Antibiotic Resistance Database (CARD) contains higher repertoire of resistance-associated mutations which can be more beneficial in examining AMR in clinical contexts. Moreover, CARD also dominates due to the easy accessibility of its annotation tool (RGI). Therefore, the selection of appropriate databases is crucial for drawing AMR-related conclusions across different study contexts. A significant drawback of genomics-enabled AMR surveillance is the frequent oversight of the socioeconomic factors that influence the spread of AMR. While the frequency and type of antimicrobial usage contribute to the evolution of antimicrobial resistance (AMR), the ecological contexts in which antimicrobials are used increases the predictive power of the evolution and dissemination of AMR [164,165]. Higher prevalence of AMR is several low-income and middle-income countries, despite lower consumption of antibiotics per person supports the above notion. Many studies [14,16] found lower prevalence of AMR in countries with better demographic, healthcare, governance, freedom index, infrastructure and education. They did not find significant association between lower AMR prevalence and reduced antibiotic consumption in these countries. It was found that better governance index indirectly resulted in lower antibiotic consumption and resulting lower AMR prevalence. Another study revealed that low sanitation levels, variable access to quality healthcare and corruption were significantly associated with high prevalence of fluoroquinolones-resistant *E.coli* in blood samples. Yet, another leading study revealed lack of significant correlation between antimicrobial usage and global burden of resistance genes in sewage samples. An interesting study led by Oliver et al. [166], examined the healthy gut microbiome of 290 volunteers of the United States Department of Agriculture (USDA) Nutritional Phenotyping Study, through shotgun metagenomic sequencing. Aminoglycoside resistance accounted for the major AMR profile in these healthy patients. Resistance-conferring aminoglycoside-*O*-phosphotransferases (aph3-dprime) correlated negatively with total calories and soluble fiber intake. Higher intake dietary fibers were associated with higher abundance of *Clostridiaceae* in their gut microbiota and lower abundance of ARGs. Hence, nutritional status can regulate levels and patterns of AMR emergence. As described earlier, the gut microbiome can serve as a reservoir of ARGs that can be potentially transferred to pathogenic microbes during infection. Moreover, significant concerns have been raised over the role of unregulated consumption of probiotics, especially its use as a part of the antibiotic's regimen, emergence and evolution of gut resistome [167]. While genetic determinants may code for mechanisms driving AMR, much of its transmission is significantly impacted by these extrinsic socioeconomic factors and dietary habits that can be monitored within the One Health framework. A comprehensive elucidation of the complex interplay between socioeconomic variables along with health and nutrition indices is beyond the scope of the review and has been extensively reviewed elsewhere [14,15,168].

### 4.6 Future directions

Clinical susceptibility to antibiotics is primarily determined by assays against pre-defined and limited bacterial population. Varying bacterial load at the site of infection, diversity of resident microbes can strongly affect antibiotic response patterns, thus, affecting treatment regime [169]. Moreover, local bioavailability of the antibiotic will vastly vary across tissue sites which will lead to formation of tissue-specific local resistance patterns [170].

Genomics has immensely spurred the detection of clinically relevant pathogenic strains that are associated with AMR. By temporal and spatial sampling of pathogens from human populations, it can be used to form an evolutionary map that delineates the plausible trajectory of pathogen fate, especially with regards to developing resistance against currently used antimicrobials [171,172]. Significant research has been dedicated to assess geographical, socio-economic and environmental factors including frequency and type of antibiotic consumption, which can be used to forecast patterns of AMR emergence. Most studies focus on upregulation and acquisition of resistance-associated genetic variation as a measure of resistance capacity of pathogens. Mortality outcomes associated with presence of such mutations targeting specific antibiotics should be included in mathematical modelling underlying prediction pipelines. This is essential since all genetic mutations in AMR patients may not equally contribute to disease outcomes and efforts should be consolidated towards targeting specific genes associated with increased mortality.

Emergence of AMR is not exclusively determined by human antibiotic consumption. Pathogens can acquire resistance elements from their surrounding contaminated soil, water and as well as other animal hosts which can eventually affect human microbial resistance profiles through the food chain. Hence, temporal and spatial sampling of environmental including veterinary and food microbial population is also crucial in predicting evolution of AMR landscape in pathogens affecting humans.

Pathogen evolution across host population is also critically regulated by individual immune response and community immunity status [173,174]. Acquisition of resistance-conferring during evolution can also have significant impacts on the adaptive fitness of microbes. The relative fitness of a resistant pathogen determines its rates of persistence and transmission. Some resistance phenotypes incur significant damage to pathogen fitness, rendering them incapable of widespread transmission. Resistant pathogens that have maintained their fitness can readily adapt to heterogenous niche (open genomes or generalists) resulting in the emergence of international clones, characteristic of widespread geographical dissemination. Hence, monitoring of AMR also requires specific distinction and identification of ‘fitter’ clones that possess the potential of epidemic and pandemic outbreaks. Rapid virulent pathogen evolution positively correlates with attenuated immune response. Evaluation of immune status of an individual or a community, and its genetic determinants might provide acute insights into curbing evolution of AMR. Monitoring vaccination status, past-infections and associated drug treatments, analysis of the dietary habits and affected commensal microbiome, and tissue-specific sampling of pathogenic microbes, can be a promising strategy to overcome barriers associated with AMR treatment. Sensitive and accurate AMR prediction at the individual level can be used to formulate drug regimens for successful treatment while preventing development of novel resistance. Single cell microbial sequencing was primarily hindered by technical limitations such as low mRNA abundance, lack of polyadenylation and difficulty in lysing cell walls. Recent advances in the development of single cell microbial transcriptomics like microSPLiT [175], ProBac-seq [176] and M3-seq [177] will prove to be useful in the detection of rare microbial states including acquisition of AMR in a population.

The robustness of NGS methodologies, encompassing genome assemblers, database and in-silico-based detection of existing or novel AMR genetic determinants, is indispensable for the successful integration of genomics surveillance into routine clinical applications. A significant step in this direction was made by Sherry et al. [178], who developed an ISO-certified computational platform called abritAMR for the genomics-enabled detection of AMR genes. Through validation across 1500 different bacteria and 415 resistance genes, this platform demonstrated a remarkable accuracy and specificity of approximately 100 % and a sensitivity of nearly 98 %.

Mendes et al. [179] spearheaded a commendable initiative aimed at establishing a standardized approach for AMR surveillance across clinical and public health domains. The Public Health Alliance for Genomic Epidemiology (PHA4GE) along with 17 public health laboratories spanning 10 countries, constructed a cohesive bioinformatic tool with standardized outputs for AMR detection in microbial genomes. The variability in efficacy among existing AMR detection tools stems from differences in reference databases, search algorithms, and default parameters. Moreover, these tools yield outputs in diverse non-standard formats and employ varied terminologies and interpretation guidelines, posing a considerable challenge for comparison and integration into analysis workflows. “HAMRonization” serves as a unified specification standard achieved through the comparison and consolidation of outputs from 18 existing detection tools. Embracing this standardized output format enhances the compatibility of AMR surveillance workflows, facilitating comparison of results across different research settings and public health sector.

Numerous global efforts are underway to curate phenotypic data on antimicrobial resistance (AMR) and to establish unified databases and repositories for worldwide surveillance [180,181] One such initiative is Pfizer's Antimicrobial Testing Leadership and Surveillance (ATLAS) program, which offers an open-access database containing anonymized raw patient minimum inhibitory concentrations (MICs) and associated clinical metadata. This program collaborates with the International Health Management Associates (IHMA), where participating laboratories send up to 280 isolates annually for phenotypic antimicrobial susceptibility testing (AST) and standard interpretation. Another significant initiative is the Global Antimicrobial Resistance and Use Surveillance System (GLASS) by the World Health Organization (WHO). GLASS works with countries that have existing national surveillance programs. Participating nations submit AMR data to GLASS, adhering to AST interpretation guidelines from the Clinical & Laboratory Standards Institute (CLSI), the European Committee on Antimicrobial Susceptibility Testing (EUCAST), or the U.S. Food and Drug Administration (FDA). GLASS then analyzes, validates, and reports the data on an annual basis. The WHO Collaborating Center for Surveillance of Antimicrobial Resistance has developed WHONET, a free Windows-based software to help microbiology laboratories analyze susceptibility test results [182]. WHONET features an automated platform that simplifies the transfer of raw susceptibility data from laboratory systems to surveillance systems like GLASS using a data conversion tool [182]. Additionally, WHONET is expanding its efforts to include the curation of AMR data from food and animal sources. Over 2300 laboratories across 130 countries have adopted WHONET as a centralized database of AMR (https://whonet.org/).

To enhance these efforts, linking comprehensive genomic databases with epidemiological and clinical metadata is essential. Whole genome sequencing (WGS) data should represent diverse geographical distribution, including both high-income countries and low- and middle-income countries (LMICs), to provide a complete picture of AMR prevalence. Establishing international standards for uniform protocols, bioinformatics algorithms, and software for species and strain identification, as well as for determining the association between mutations and resistance phenotypes, is critical. Uploading raw sequence data along with quality assurance reports will facilitate comparison and accurate interpretation. As a part of these global initiatives, integrating WGS data for a subset of priority pathogens and organisms reported under the Emerging Antimicrobial Resistance Reporting system could initially be implemented to predict AMR trends effectively (GLASS Whole-genome sequencing for surveillance of antimicrobial resistance, WHO, 2022). Effective curation and integration of phenotypic AST and WGS data, along with epidemiological information, can help identify high-risk clones, at-risk populations, and regions prone to AMR outbreaks by tracing transmission networks. As previously explained, endeavors to standardize and enhance the throughput capabilities of existing next-generation sequencing (NGS) technologies and bioinformatics methodologies coupled with provisions of open-access universal databases, computational platforms, and standard interpretation guidelines, need to be closely integrated into the One Health framework. This is vital for ensuring easy access to and integration of genomic data within the public health sector, enabling healthcare workers, researchers, and policymakers to implement guidelines and strategies for nationwide AMR monitoring. Additionally, this facilitates the incorporation of national data into global surveillance efforts and the prediction of AMR trends.

## 5 Conclusion

Global surveillance of AMR, hence, requires an integrated genomic-epidemiologic approach involving widespread geographical as well as local sampling [65]. Complex inter-relationships between all biotic and abiotic contributors of this silent yet pervasive health concern needs to be delineated, so as to enable its rapid prediction and resolution. Genomic surveillance allows for the empirical characterization of resistance profiles as specific repertoires of resistance-conferring genetic determinants and their genetic context, whose patterns of emergence and distribution can be tracked over space and time. Incorporating this surveillance into the One Health strategy is crucial, as it facilitates a highly definite, multiparametric analysis of growing AMR risks around the globe.

### 5.1 Study limitations

This review aims to highlight recent findings on the application of next-generation sequencing for antimicrobial resistance surveillance and their potential contributions to the One Health framework of AMR surveillance. Due to length constraints and availability of extensive research in this field, it does not cover genomic findings related to every microorganism or infection. Instead, we focus on few key discoveries and concepts that illustrate the current status and applications of NGS and AI-enabled approaches in AMR surveillance. This review is not intended to be a comprehensive evaluation or description of all progress made in genomics for AMR detection and prediction. We primarily concentrate on AMR trends and NGS applications to bacterial pathogens. This focus, however, does not diminish the importance of addressing viral, fungal, and parasitic AMR. Additionally, while we concentrate on NGS findings, we do not delve into the complex interplay of other crucial factors influencing AMR trends and emergence, such as socioeconomic factors, development and sanitation, antibiotic usage, and public health and nutrition status. Although not discussed in this review, the importance of education and public outreach is critical for the One Health approach to understanding AMR [183].

## 6 Box 1

### 6.1 Pathogen evolution: prelude to antimicrobial resistance

Pathogen evolution is a dynamic process whose trajectory and fate is determined by both pathogen-intrinsic and extrinsic or host-specific factors [184,185]. Evolution of pathogens is vital for their survival, fitness and propagation within and across the host populations. Pathogens which accumulate critical mutations that enhance their capacity to establish infections and subsequent transmissions become causative agents for an epidemic/pandemic outbreak. Stringent regulation of pathogen virulence is crucial for optimal infectivity and transmissibility. Apart from pathogen-intrinsic modules of virulence determinants, host response to pathogens also mold pathogen virulence [[186], [187], [188]]. Immunocompromised individuals will experience higher virulence of the same pathogen compared to a healthy individual. Moreover, immune memory to pre-exposure to pathogens will also consequently attenuate virulence [173]. A pathogen's evolutionary capacity is determined by its ability to mutate and evade host immune surveillance. Every mutational adaptation incurs a significant fitness cost which limits its evolutionary trajectory [189]. Increased host diversity, therefore, results in diminished likelihood of pathogen emergence and evolution [190,191].

A major consequence of pathogen evolution that poses a formidable global threat is the emergence of antimicrobial resistance (AMR) which is considered to be a ‘silent pandemic’ [192]. A global estimate in 2019 revealed a staggering toll, with 4.95 million deaths attributed to AMR. Among these, 1.27 million deaths are directly caused by the bacterial AMR. Alarmingly, one out of five children under the age of 5 succumbs to treatable infections as a consequence of non-responsiveness to current antibiotics (World Health Organization. 2023). Mortality associated with AMR has surpassed those caused by HIV, malaria and tuberculosis combined. This strongly suggests that research dedicated to identification of environmental, social and genetic factors contributing to this unimpeded process should be a pressing priority.

## 7 Box 2

### 7.1 Evolution of drug-induced resistance in microbes

The prevailing notion that AMR is a modern phenomenon, is largely fueled by the observation that microbes which were found before the antibiotic era were highly susceptible to antibiotics. However, the discovery of a large repertoire of AMR genes through metagenomics studies of ancient permafrost samples, challenged this perspective [193]. These findings strongly suggest that antimicrobial resistance is natural and inevitable. The unprecedent rise in AMR cases during the modern era, could however, be attributed to enhanced evolutionary pressure originating from urban development and uncontrolled use of antibiotics [26].

Efforts to combat global drug resistance are concentrated on development of newer drugs or targeting susceptible variants. These approaches, however, do not abolish the origin and driver of resistance – evolution of microbes in response to selection pressure. Evolution of AMR is a multifaceted phenomenon with complex interactions between various determinants, including rate of resistance mutations, fitness benefits conferred by the mutation on the pathogen's survival, duration/extent of selection pressure, level of resistance developed as well as coinfections with multiple strains [194,195]. Other factors include epistatic interactions between genes and compensatory mutations so as to maintain fitness, development of resistance to more than one class of drugs, and co-selection of genes which are linked with antibiotic resistance [196,197]. All these components form a highly intricate network which makes prediction of microbial evolution extremely challenging. AMR prevalence in infectious and non-infectious diseases are described in Box 3. Mechanisms driving AMR are described in Box 4 and illustrated in Fig. 1, and reviewed extensively elsewhere [194,[198], [199], [200]].

The gut microbiome presents as a significant anomaly to the prevailing notion that exposure to antimicrobial drugs drive evolution of pathogen resistance. The gut microbiome comprises a unique environment of co-existence of commensals, pathogens and high antibiotic selection pressure [201]. The homeostatic gut microbiome produces a vast repertoire of antimicrobial peptides (AMPs) that plays a significant role in maintaining balance of resident commensal species while maintaining gut immunological tolerance along with protection from enteric diseases [202]. Research has demonstrated that antibiotics can disrupt the gut microbiome by selectively eliminating certain commensal populations while allowing others to proliferate. This disruption negatively impacts the integrity of the mucus barrier, potentially leading to breaches in immune tolerance as segregated microbes are systemically released [203]. While many studies have characterized commensal microbiota as long-term reservoirs of AMR, a significant body of research have revealed synergistic antimicrobial action of antibiotic and microbiome-produced AMPs. Antibiotic-induced gut dysbiosis creates a strong survival competition between disrupted species populations [204]. Together with high concentrations of external antibiotics and resident AMPs, the gut microenvironment imposes a formidable selection pressure which can potentially foster development of resistance [201]. Given the intense evolutionary pressure, it is rather remarkable that gut-resident microbes have retained their capacity to produce AMPs or have not diminished the efficacy of these peptides. This can be significantly attributed to the intrinsic diversity of microbiome AMPs and also their diverse modes of antimicrobial action. AMPs exert their function through multiple mechanism including membrane pore formation, inhibition of cell wall, RNA, DNA, protein synthesis and folding, biofilm disruption and inhibition of spore formation [201]. Evolution of resistance against multiple targets would warrant extensive genetic evolution which could adversely impact microbial genome fitness. Hence, broad spectrum of activity, high specificity for microbial targets and low likelihood of resistance development make AMPs attractive targets for enhancing antibiotic efficacy in clinical settings [205].

## 8 Box 3

### 8.1 Antimicrobial resistance in infectious disease

Pathogens drivers of commonly occurring infections like sepsis or diarrhea, are often associated with AMR. According to the estimates from Global Antimicrobial Resistance and Use Surveillance System (GLASS 2020), AMR rates has been reported to be as high as 92.9 % for *E. coli* and 79.4 % for *K. pneumoniae* against ciprofloxacin-treatment of urinary tract infections. Carbapenems belong to a class of potent antibiotics that can treat multi-drug resistant infections. Growing resistance to carbapenems by *K. pneumonia* can therefore, cause fatal disease. ESKAPE pathogens comprising of *Enterococcus faecium*, *Staphylococcus aureus, Klebsiella pneumoniae*, *Acinetobacter baumannii*, *Pseudomonas aeruginosa* and *Enterobacter* spp – show multi-drug resistance and cause majority of nosocomial infections [206]. Multi-drug resistant TB (MDR-TB) show resistance to at least two classes of antibiotics – isoniazid and rifampicin. Less than 60 % of MDR-TB show recovery upon treatment [207].

### 8.2 Antimicrobial resistance in non-infectious diseases

An alarming trend of antimicrobial resistance-mediated unfavorable outcomes are observed in non-microbial diseases like cancer [208]. The human microbiome is essential for optimal and broad T cell priming against antigens which have a beneficial outcome in anti-cancer immune response. Positive prognosis in checkpoint blockade therapy is associated with a healthy and diverse gut microbiome [209,210]. A significant proportion of mortality in cancer patients is due to sepsis from blood infections. Chemotherapy results in undesirable effects like neutropenia and disruption of the gut barrier which causes systemic dispersal of gut microbes. Gut dysbiosis as a result of chemotherapy also promote outgrowth of pathogenic microbes. Moreover, de novo mutagenesis and accelerated HGT induced by chemotherapy can culminate in emergence of resistance against antibiotics administered to these patients [208]. Vancomycin resistance in *E. faecium* and fluoroquinolone resistance in *Escherichia coli* is predominantly associated with cancer patients undergoing chemotherapy [211,212].

### 8.3 Antimicrobial resistance due to use of non-antibiotic drugs

Studies have shown emergence of AMR upon consumption of non-antibiotic pharmaceuticals [213]. Antidepressants are consumed at similar rate of approximately 5 % compared to antibiotic consumption. Resistance was associated with induction of stress response like production or reactive oxygen species under aerobic conditions and upregulated expression of efflux pumps. Commonly prescribed antidepressants like sertraline and duloxetine trigger multidrug resistance as well as bacterial persistence in the presence of ciprofloxacin. SNPs associated with aforementioned mechanisms of resistance showed stable vertical transmission through generations [213]. Similar associations pertaining to consumption of Serotonin-norepinephrine Reuptake Inhibitor (SNRI) and Selective Serotonin Reuptake Inhibitor (SSRI) and HGT-mediated bacterial transformation, was reported earlier. Genome-wide RNA screening revealed enhanced oxidative stress, altered cell permeability and genes affecting competence, SOS response and recombination mediators of resistance [214].

## 9 Box 4

### 9.1 Pathways involving acquisition of resistance determinants

A pathogen can accumulate changes in its genome resulting in antimicrobial resistance through 2 major distinct pathways: horizontal gene transfer and accumulation of de novo mutations. These are described in detail in Refs. [198,199].

**Horizontal gene transfer:** Most clinically used antimicrobial drugs are found in the environment and there is prevalence of microbes which have intrinsic antimicrobial resistance. It has been established that this environmental reservoir is a major source of acquisition of AMR by horizontal gene transfer (HGT). HGT involves 3 major processes of genetic exchange: transformation, transduction and conjugation, reviewed elsewhere. The most efficient form of genetic exchange through HGT is conjugation and is widely prevalent in hospital-associated resistance through transfer of mobile genetic elements like plasmids and transposons. Mobile AMR elements have been associated with global spread of AMR related to b-lactamases (ESBLs) and the carbapenemases, KPC and NDM-1. Species such as *K. pneumoniae* have a high capacity for horizontal gene transfer, and can act as bridge for resistance transfer from large bacterial populations to bacterial populations associated with humans.

**De novo mutagenesis:** Stress conditions like starvation or antibiotics can lead to appearance of a subset of cells that have undergone error-prone, stress induced DNA repair which leads to accumulation of de novo mutations. Most de novo mutations confer a fitness disadvantage to the harboring population but can be maintained under selection pressure like antibiotics-mediated killing.

### 9.2 Mechanisms of antimicrobial resistance

Genes which are drivers or associated with resistance are generally target of the drugs against which resistance is developed. There are three general ways by which microbial mutagenesis can lead to development of drug resistance as described in detail in references, 198 and 200.

**Modification of target protein:** Drug resistance mutations majorly target and modify regions of protein involved in drug binding or function. Resistance to fluoroquinolones are associated with mutations in genes coding for topoisomerase function. Similarly, rifampicin (RIF) resistance in tuberculosis is associated with mutations in DNA-dependent RNA polymerase β-subunit, encoded by the rpoB gene. Mutations occurs in the rifampicin-binding site at the β subunit of the RNA polymerase which dramatically reduces affinity of rifampicin to the Rpo protein. An interesting study revealed that out of 34 mutations associated with RIF's resistance in the Rpo gene, 25 mutations impeded drug-protein structural interactions causing resistance [215]. Such in protein-drug interactions is a key mechanism contributing to AMR.

**Upregulation of export transporters:** Multi-drug resistance is a predominant occurrence in Gram negative bacterial populations and is associated with increased expression upregulation of drug efflux pumps [216]. Families of microbial efflux transporters include resistance-nodulation-division (RND) superfamily exporters, major facilitator superfamily (MFS), multidrug and toxic compound extrusion (MATE), small multidrug resistance (SMR), and ATP-binding cassette (ABC) superfamilies. While ABC transporters utilize energy generated from ATP hydrolysis, other transporters use energy generated from proton gradients across channels [216]. Drug resistance in *Mycobacterium tuberculosis* has been shown to be attributed to at least 30 efflux transporters mostly belonging to ABC and MFS superfamilies [217]. Drug efflux-driven resistance is not just found in first-line TB drugs like isoniazid, rifampicin, ethambutol, and pyrazinamide, but also in the recently approved Bedaquiline, belonging to a class of diarylquinoline drugs targeting ATP synthase [218]. Resistance was driven by upregulation of Rv0676c/Rv0677c (MmpS5/MmpL5) efflux pump [219]. Global emergence of multidrug -resistance in tuberculosis has let to adoption of a distinct strategy involving co-administration of efflux pump inhibitors (EPI) along with antibiotics to combat resistance [220]. However, this strategy has not met with significant success since no clinically approved EPI exists currently.

**Tolerance to antibiotics:** The process of resistance to antibiotics is distinct from that of tolerance to antibiotics [221,222]. Antibiotic resistance is determined by elevated minimum inhibitory concentration (MIC) which is the measure of the lowest antibiotic concentration that will achieve effective pathogen killing. Antibiotic tolerance, however, has no effect on MIC. Tolerant microbes often survive in very high bactericidal antibiotic doses. Tolerant pathogens generally show a lower death rate at similar antibiotic concentrations compared to susceptible populations. Minimum duration of killing (MDK) is a measure of microbial tolerance to antibiotics to denote the length of time, a tolerant population can withstand antibiotics-mediated killing. Microbial tolerance is associated with slower growth rate under antibiotic pressure, unlike resistance which can resume growth even in higher concentrations of antibiotics. Unlike resistance, if tolerance occurs in a subset of microbial population, these tolerant cells tend to persist and survive under environmental stress. Resistance occurring in a subset of cells, however, tend to be lost in the absence of external antibiotic selection pressure.

## 10 Box 5

### 10.1 Current strategies for combating drug-induced resistance in microbes

Drugs that target antibiotic-induced mutagenesis are being currently evaluated. These ‘evolution-slowing’ drugs impede microbial evolution in response to stress like antibiotics, which could principally prolong the efficacy of antibiotics [223,224]. Significant research has been performed that identify potential microbial targets for these ‘evolution-slowing’ drugs. Mamun et al. identified a network of ≥93 genes that were associated with mutagenesis [225]. Stress-inducing factors like starvation, oxidative stress or antibiotics form the major component of mutagenesis and evolution of microbes [226,227]. Elucidating a detailed map of the mutational and evolutionary landscape of microbes can help predict regions of microbial genomes susceptible to mutation, and consequently, their propensity to develop specific drug resistance [228,229]. Preliminary in-vitro studies to combat resistance to fluoroquinolones, especially ciprofloxacin, have identified dequalinium chloride (DEQ) as potential ‘evolution-slowing’ drug [230]. Ciprofloxacin follows beta lactams as the second-highest prescribed antibiotic and its resistances is acquired mostly through de novo mutations that alter target protein, prevent binding to target protein and upregulation of drug-efflux channels [231]. Mutagenesis in target bacteria is mostly induced through antibiotic-mediated activation of stress response pathway. DEQ inhibits σ^S^, the central mediator of stress response, thereby impeding evolution of resistance [227]. Synthesis of novel ‘evolution-slowing’ drugs can potentially eliminate the need of antibiotics altogether, if evolution of pathogen can be impeded to an extent which allows its complete eradication by the immune system. This approach has an added advantage of preserving the commensal microbial community in the host which is largely affected by general antibiotic consumption [230,232].

Many studies have proposed the use of antibiotic adjuvants that are compounds that lack any intrinsic antimicrobial activity [233]. They are however, administered along with target antibiotic where they execute synergistic action of blocking resistance development and enhancing antibiotic efficacy. One notable example includes beta-lactams (antibiotic) and beta-lactamase (antibiotic resistance) inhibitors. Antibiotic adjuvants can act directly on pathogens by blocking resistance (Class I adjuvants) or can modify host response to enhance drug efficacy (Class II adjuvants) [234]. Major functional classes of Class II adjuvants include β-lactamase inhibitors, efflux pump inhibitors (EPIs), quorum quenchers, proton motive force (PMF) inhibitors and membrane permeabilizers [234]. As described earlier, there is also a growing reserve of research dedicated to structural and biochemical modifications of natural AMPs so as to improve their bioavailability and half-lives. This will facilitate their use in clinical contexts as replacements or adjuvants of sub-optimal antibiotics that are subjected to resistance [204]. Antibiotic adjuvants have been under considerable research and use for more than three decades, proving to be invaluable in revitalizing existing drugs rendered ineffective by resistance [235].

Other researchers have attempted to implement drug-free strategies to curb AMR. Plackett provides a concise summary of three recent developments in strategies for mitigating AMR [236]. Preliminary studies have demonstrated considerable efficacy of plasma water as a potential replacement of conventional antimicrobial disinfectants, particularly in methicillin-resistant *Staphylococcus aureus* (MRSA). Plasma water contains free radicals like reactive oxygen and nitrogen species. They induce considerable oxidative stress-mediated in DNA damage and resulting microbial death. Another promising avenue of research is use of metals like gallium in the disruption of bacterial biofilms which promote bacterial survival in chronic human infections. Drugs containing gallium are being currently being explored and have been shown to disrupt MRSA biofilms and bacterial killing at about one-tenth of the usual antibiotic dose. The third strategy employs computer simulations of the outer bacterial wall to assess the permeability of existing compound libraries. By training and simulations with these compounds, researchers have explored the structural attributes that can be potentially modified to enhance compound permeability. There is a growing trend of using bacteriophage therapies to combat antimicrobial resistance (AMR) [237]. Bacteriophages typically reproduce in two ways: lytic and lysogenic cycles. Lysogenic bacteriophages infect host bacteria and integrate their viral genome as a prophage, allowing their DNA to replicate without killing the host. These phages remain dormant in the host bacteria until specific signals trigger activation. Lysogenic prophages pose a significant threat to AMR by increasing the likelihood of AMR gene transfer from host bacteria to other populations. Conversely, lytic bacteriophages hijack the bacterial replication machinery to replicate their viral DNA and eventually lyse the host cell to release new viral progeny. Lytic bacteriophages effectively kill and eliminate bacteria and can be engineered to target specific strains and species. As bacteriophages adapt and evolve with bacterial communities, they can be successfully employed to combat resistant microbes [238].

While the aforementioned strategies show potential in overcoming existing AMR, the primary focus should be concentrated on efforts at reducing the emergence and evolution of AMR. This entails thorough monitoring of the spatiotemporal patterns of AMR evolution and transmission, which would enable the accurate prediction of AMR risks and development of guided strategies for its containment.

## 11 Box 6

Genomic epidemiology involves mass sequencing of pathogen genomes across spatially diverse populations, which enables real time tracking of pathogen transmission and its genome evolution [239]. Genomic epidemiology is a powerful tool to predict local and global infection outbreaks and consequently facilitates development of strategies for identification, prevention and/or restriction of such outbreaks. It can be used to evaluate the emergence of novel pathogens with potentially enhanced transmission capabilities or acquired resistance to treatments. The West African Ebola pandemic was one of the first major applications of this tool, wherein mass viral genome sequencing revealed origin and spread of this outbreak [[240], [241], [242]]. The recent SARS-CoV-2 pandemic was also saw a widescale use of this tool to delineate and predict disease trajectories which guided public health policies for its containment and treatment [243,244]. Despite being limited to observational data and lack of experimental validation, advanced mathematical and computational modelling has created an effective framework to perform simulate trajectories of pathogen evolution across fitness valleys (stochastic tunneling), selection, transmission and potential outbreaks [245].

Epidemic outbreaks potentially rising from local contaminated sources requires identification of those sources for future surveillance as well as containment. Food borne pathogens like *Salmonella* and *Listeria monocytogenes* form a major source of such local epidemics. Genomic epidemiology allows monitoring of such pathogen transmissions and emergence of novel and/or drug resistant strains which is crucial for their prevention. By genome sequence typing of microbes and evaluating their phylogenetic relationships, once can identify potential clusters and trace their origin [246].

## Glossary

**Antimicrobial resistance:** AMR refers to the inherent ability of microbes, including bacteria, viruses, fungi, and parasites, to withstand the inhibitory or killing effects of antimicrobial drugs. This resistance leads to significant mortality from infections that are otherwise treatable.

**Multidrug resistance:** MDR occurs when a microorganism exhibits resistance to multiple classes of antibiotics, each with distinct mechanisms of action. This poses a considerable challenge as infections become unresponsive to conventional antibiotic treatments.

**One Health:** One Health is an integrative approach that focuses on improving the three pillars of global health: human, animal, and environmental health. This approach aims to foster cooperation among various sectors, including public health, environmental science, veterinary medicine, and agriculture, to prevent disease transmission (including zoonotic diseases) and promote the sustainability of ecological health.

**Minimum inhibitory concentration:** MIC is the lowest concentration of an antimicrobial drug that inhibits the growth of the targeted microorganism. Clinical breakpoints are specific MIC values used to classify a microorganism as resistant, intermediate, or susceptible. If the MIC exceeds the resistance breakpoint, the microorganism is classified as resistant to the drug. Conversely, if the MIC falls below the susceptible breakpoint, the microorganism is considered susceptible to inhibition or killing by the drug.

**Next generation sequencing:** Next-generation sequencing (NGS) is a high-throughput technology that enables the rapid parallel sequencing of DNA and RNA. It offers a cost-effective means for comprehensive analysis of genomes and transcriptomes, providing greater accuracy and sensitivity compared to traditional Sanger sequencing.

**Whole genomic sequencing:** WGS enables sequencing of the entire genome including chromosomes and mobile genetic elements of a single organism. This process requires isolating the specific microorganism from the source sample and often involves prior culturing.

**Metagenomic sequencing:** Metagenomic sequencing examines the combined genomes of all microbial isolates within a community directly from environmental samples.

**High-throughput Chromosome Conformation Capture:** The Hi-C technique is employed to examine the three-dimensional (3D) arrangement of microbial genomes. It begins with the cross-linking of proximal chromatin regions in their natural 3D configuration. Following fragmentation, ligation, sequencing, and computational analysis, researchers can determine the spatial context of elements like AMR genes or genetic components such as their positioning on chromosomes or within mobile genetic elements (MGEs). Analyzing the spatial organization of AMR genes can provide insights into their phylogenetic origins, evolutionary patterns and transmission dynamics.

**Microbiome:** The host microbiome encompasses all microbial species residing within specific anatomical niches of the host, such as the skin, gut, and mucosa of the respiratory and reproductive tracts. These microbes play symbiotic roles in supporting various physiological and immune functions of the host. The microbiome can consist of purely beneficial commensals or opportunistic pathobionts. Pathobionts are commensals that reside within the host microbiome but can become pathogenic under conditions of inflammation-induced dysbiosis (disruption of microbial community structure), loss of barrier integrity and invasion into the blood stream.

**Resistome:** The host resistome encompasses all genetic elements responsible for conferring resistance to various antibiotics within the diverse microbial populations, both commensal and pathogenic, inhabiting the host. This includes both established and newly identified or potential antimicrobial resistance genes.

**Epidemiology:** The field of epidemiology encompasses the examination of distribution and patterns of health-related events and diseases within and across populations. This involves a systematic investigation of the factors that influence the occurrence and transmission of diseases, such as their causes, risk factors, modes of transmission, and outcomes.

**Genomic epidemiology:** Genomic epidemiology employs genomic sequencing techniques to investigate the spread and transmission dynamics of infectious diseases within and across populations.

**Phylodynamics:** Phylodynamics integrates principles and methodologies from both phylogenetics, which examines evolutionary relationships based on genomic data, and epidemiology. It entails analyzing genetic sequences from microbial samples collected over time and across geographic areas. By doing so, it reconstructs the evolutionary trajectory of a microorganism within a population and infers its transmission rates and patterns of geographical spread, based on epidemiological data.

**Polygeography:** The polygeography of a pathogen describes its geographic distribution spanning multiple regions, influenced by factors like migration, trade, and ecological conditions. This aspect is pivotal for conducting epidemiological surveys of AMR outbreaks.

**Specialist microbes:** Specialist microbes have undergone rigorous evolutionary processes to acquire distinct genetic traits, metabolic pathways, or physiological adaptations that enable them to thrive in specific environmental conditions.

**Generalist microbes:** Generalist microbes exhibit the ability to thrive in diverse environmental conditions and habitats, displaying extensive ecological tolerance. Their remarkable genetic, metabolic, and physiological adaptability enables them to efficiently inhabit various hosts and environments.

**Pangenome:** The pangenome encompasses the entire complement genes present across all members of a species. This includes both the core genome, consisting of genes shared by every individual within the species, and the accessory genome, which comprises genes unique to specific subsets of individuals.

**Artificial intelligence:** AI involves computers and machines simulating human intelligence. This encompasses learning and reasoning from input data, problem-solving, perception, and interaction with the environment.

**Machine learning and deep learning:** ML and DL are components of AI dedicated to designing algorithms and models that enable computers to learn from input data and make decisions or prediction. ML involves training algorithms on labeled datasets, where inputs are paired with corresponding targets or outputs and subsequent adjustment of parameters for optimization of the model's performance. On the other hand, DL utilizes multi-layered neural networks to learn from extensive datasets. DL is proficient at automatically discerning intricate patterns in complex data and finds applications in areas such as image recognition, speech processing, and natural language processing.

**Supervised learning:** SL is an approach in machine learning where algorithms are trained on datasets containing paired inputs and their corresponding outputs (targets). This methodology aims to map the inputs to their respective outputs accurately for predictions or classifications. During training, SL algorithms adjust model parameters to minimize differences between predicted and actual (target) outputs using various optimization techniques. Examples of SL algorithms include logistic regression, support vector machines, decision trees, and random forests, where genomic features serve as inputs mapped to labeled target outputs representing resistance phenotypes. These algorithms are utilized to train models on labeled data, enabling predictions of the likelihood of a microbe being resistant or susceptible to a specific antibiotic.

**Neural networks:** Neural networks play a crucial role in deep learning (DL) models for predicting antimicrobial resistance (AMR). By training on labeled data through SL, neural networks can effectively classify microbial strains as either resistant or susceptible. Moreover, neural networks can be utilized to predict quantitative measures of AMR, such as minimum inhibitory concentrations (MICs), using both genotypic and phenotypic data. They have the capability to capture intricate hierarchical patterns and networks associated with AMR, which can be employed as input features for downstream classification or regression tasks. Additionally, neural network models can be applied in transfer learning (TL), where neural network models trained and optimized on large datasets can be employed to predict and classify outcomes in smaller and imbalanced datasets.

**Confusion matrix:** A confusion matrix serves as a tool for assessing the effectiveness of a classification model. It offers a concise overview of the model's predictions in comparison to the actual outcomes across various categories. By considering true positive (TP), true negative (TN), false positive (FP), and false negative (FN) values, the confusion matrix aids in the computation of performance metrics like precision, recall, accuracy, and F1-scores.

**Multilabel classification:** In contrast to conventional binary classification, which focuses on distinguishing between resistant and susceptible phenotypes for a single drug, multilabel classification (MLC) concurrently predicts and categorizes resistance or susceptibility across multiple drugs. MLC allows for the classification of each isolate based on its response to multiple antibiotics, where an isolate may be susceptible to one antibiotic while resistant to another, or resistant to multiple antibiotics.

**Ensemble classifier chain:** This model is a subset of multilabel classification (MLC) and is designed for the simultaneous prediction of antimicrobial resistance (AMR) to multiple antibiotics. Employing a chain of classifiers, each model is trained to predict the resistance or susceptibility of a single microbe to a specific drug. The output from each classifier within the ensemble serves as input for subsequent classifiers. Leveraging diverse classifiers enables the elucidation of complex relationships among multidrug resistance profiles, ultimately enhancing the accuracy of AMR prediction.

**Random subspace ensembles:** It is a ML technique of classification where multiple models or base classifiers are trained on randomly selected, distinct subsets of features of original input data (genomic sequence). The consequent diversity introduced facilitates capture of different aspects of the data. The output predictions of each classifier model are combined to produce the final ensemble prediction. By harnessing the diversity inherent in each feature subset, it enhances the overall adaptability and robustness of the model.

**Bayesian network:** It is a graphical model consisting of nodes representing variables such as genetic determinants of AMR, antibiotic consumption, patient clinical data, environmental conditions. Directed edges connecting the nodes represent probabilistic connections and dependencies among these variables. This framework adequately captures intricate interactions among diverse factors influencing the probability of AMR emergence and dissemination. The process involves iterative resampling of the initial dataset to create numerous data subsets known as bootstrap replicates. Each replicate contains the same number but different combinations of observations. For every bootstrap sample, a Bayesian network is constructed from the data, capturing the probabilistic dependencies among variables. Analyzing a substantial number of bootstrap samples allows for evaluating the variability in the learned networks. This approach significantly improves robustness, accuracy and generalizability of the model.

## Funding

CSCL acknowledge the funding support from 10.13039/100000865Bill and Melinda Gates Foundation (BMGF), Grant number - INV-033578, for her salary.

## CRediT authorship contribution statement

**Chinky Shiu Chen Liu:** Writing – original draft, Visualization, Investigation, Formal analysis. **Rajesh Pandey:** Writing – review & editing, Supervision, Project administration, Funding acquisition, Conceptualization.

## Declaration of competing interest

The authors declare that they have no known competing financial interests or personal relationships that could have appeared to influence the work reported in this paper.

## References

[1] Larsson D.G.J., Flach C.F. (2022). Antibiotic resistance in the environment. Nat. Rev. Microbiol..

[2] Toner E., Adalja A., Gronvall G.K., Cicero A., Inglesby T.V. (2015 May-Jun). Antimicrobial resistance is a global health emergency. Health Secur.

[3] Prestinaci F., Pezzotti P., Pantosti A. (2015). Antimicrobial resistance: a global multifaceted phenomenon. Pathog. Glob. Health.

[4] James C., James S.J., Onarinde B.A., Dixon R.A., Williams N. (2023 Oct 27). A critical review of AMR risks arising as a consequence of using biocides and certain metals in food animal production. Antibiotics (Basel).

[5] Joshi M.P., Alombah F., Konduri N., Ndiaye A., Kusu N., Kiggundu R., Lusaya E.P., Tuala Tuala R., Embrey M., Hafner T., Traore O., Mbaye M., Akinola B., Namburete D., Acho A., Hema Y., Getahun W., Sayem M.A., Nfor E. (2023 Apr 14). Moving from assessments to implementation: promising practices for strengthening multisectoral antimicrobial resistance containment capacity. One Health Outlook.

[6] McEwen S.A., Collignon P.J. (2018). Antimicrobial resistance: a one health perspective. Microbiol. Spectr..

[7] Aslam B., Khurshid M., Arshad M.I., Muzammil S., Rasool M., Yasmeen N., Shah T., Chaudhry T.H., Rasool M.H., Shahid A., Xueshan X., Baloch Z. (2021 Nov 25). Antibiotic resistance: one health one world outlook. Front. Cell. Infect. Microbiol..

[8] King L.J., Anderson L.R., Blackmore C.G., Blackwell M.J., Lautner E.A., Marcus L.C., Meyer T.E., Monath T.P., Nave J.E., Ohle J., Pappaioanou M., Sobota J., Stokes W.S., Davis R.M., Glasser J.H., Mahr R.K. (2008 Jul 15). Executive summary of the AVMA one health initiative task force report. J. Am. Vet. Med. Assoc..

[9] Velazquez-Meza M.E., Galarde-López M., Carrillo-Quiróz B., Alpuche-Aranda C.M. (2022 Mar). Antimicrobial resistance: one health approach. Vet. World.

[10] Marais B., Crawford J., Iredell J., Ward M., Simpson S., Gilbert L., Griffiths P., Kamradt-Scott A., Colagiuri R., Jones C., Sorrell T. (2012 Sep 1). One world, one health: beyond the millennium development goals. Lancet.

[11] Mackenzie J.S., Jeggo M. (2019 May 31). The one health approach-why is it so important?. Trav. Med. Infect. Dis..

[12] Adisasmito W.B., Almuhairi S., Behravesh C.B., Bilivogui P., Bukachi S.A., Casas N., Cediel Becerra N., Charron D.F., Chaudhary A., Ciacci Zanella J.R., Cunningham A.A., Dar O., Debnath N., Dungu B., Farag E., Gao G.F., Hayman D.T.S., Khaitsa M., Koopmans M.P.G., Machalaba C., Mackenzie J.S., Markotter W., Mettenleiter T.C., Morand S., Smolenskiy V., Zhou L., One Health High-Level Expert Panel (OHHLEP) (2022 Jun 23). One Health: a new definition for a sustainable and healthy future. PLoS Pathog..

[13] Robinson T.P., Bu D.P., Carrique-Mas J., Fèvre E.M., Gilbert M., Grace D., Hay S.I., Jiwakanon J., Kakkar M., Kariuki S., Laxminarayan R., Lubroth J., Magnusson U., Thi Ngoc P., Van Boeckel T.P., Woolhouse M.E. (2016 Jul). Antibiotic resistance is the quintessential One Health issue. Trans. R. Soc. Trop. Med. Hyg..

[14] Larsson D.G.J., Gaze W.H., Laxminarayan R., Topp E. (2023 May). AMR, one health and the environment. Nat. Microbiol..

[15] Collignon P., Beggs J.J., Walsh T.R., Gandra S., Laxminarayan R. (2018 Sep). Anthropological and socioeconomic factors contributing to global antimicrobial resistance: a univariate and multivariable analysis. Lancet Planet. Health.

[16] Collignon P., Beggs J.J. (2019 Jun 30). Socioeconomic enablers for contagion: factors impelling the antimicrobial resistance epidemic. Antibiotics (Basel).

[17] Maugeri A., Barchitta M., Puglisi F. (2023). Socio-economic, governance and health indicators shaping antimicrobial resistance: an ecological analysis of 30 european countries. Glob. Health.

[18] Jauneikaite E., Baker K.S., Nunn J.G., Midega J.T., Hsu L.Y., Singh S.R., Halpin A.L., Hopkins K.L., Price J.R., Srikantiah P., Egyir B., Okeke I.N., Holt K.E., Peacock S.J., Feasey N.A., SEDRIC Genomics Surveillance Working Group (2023 Dec). Genomics for antimicrobial resistance surveillance to support infection prevention and control in health-care facilities. Lancet Microbe.

[19] Djordjevic S.P., Jarocki V.M., Seemann T. (2024). Genomic surveillance for antimicrobial resistance — a One Health perspective. Nat. Rev. Genet..

[20] Llor C., Bjerrum L. (2014 Dec). Antimicrobial resistance: risk associated with antibiotic overuse and initiatives to reduce the problem. Ther. Adv. Drug Saf..

[21] Sommer M.O.A., Munck C., Toft-Kehler R.V., Andersson D.I. (2017 Nov). Prediction of antibiotic resistance: time for a new preclinical paradigm?. Nat. Rev. Microbiol..

[22] Kowalska-Krochmal B., Dudek-Wicher R. (2021 Feb 4). The minimum inhibitory concentration of antibiotics: methods, interpretation, clinical relevance. Pathogens.

[23] Chowdhury A.S., Call D.R., Broschat S.L. (2019 Oct 9). Antimicrobial resistance prediction for gram-negative bacteria via game theory-based feature evaluation. Sci. Rep..

[24] Stewart E.J. (2012 Aug). Growing unculturable bacteria. J. Bacteriol..

[25] Vasala A., Hytönen V.P., Laitinen O.H. (2020 Jul 15). Modern tools for rapid diagnostics of antimicrobial resistance. Front. Cell. Infect. Microbiol..

[26] Purushothaman S., Meola M., Egli A. (2022 Aug 30). Combination of whole genome sequencing and metagenomics for microbiological diagnostics. Int. J. Mol. Sci..

[27] Baker S., Thomson N., Weill F.X., Holt K.E. (2018 May). Genomic insights into the emergence and spread of antimicrobial-resistant bacterial pathogens. Science.

[28] Balloux F., Brønstad Brynildsrud O., van Dorp L., Shaw L.P., Chen H., Harris K.A., Wang H., Eldholm V. (2018 Dec). From theory to practice: translating whole-genome sequencing (WGS) into the clinic. Trends Microbiol..

[29] Hendriksen R.S., Bortolaia V., Tate H., Tyson G.H., Aarestrup F.M., McDermott P.F. (2019 Sep 4). Using genomics to track global antimicrobial resistance. Front. Public Health.

[30] Waddington C., Carey M.E., Boinett C.J., Higginson E., Veeraraghavan B., Baker S. (2022 Feb 16). Exploiting genomics to mitigate the public health impact of antimicrobial resistance. Genome Med..

[31] Su M., Satola S.W., Read T.D. (2019 Feb 27). Genome-based prediction of bacterial antibiotic resistance. J. Clin. Microbiol..

[32] Wensel C.R., Pluznick J.L., Salzberg S.L., Sears C.L. (2022 Apr 1). Next-generation sequencing: insights to advance clinical investigations of the microbiome. J. Clin. Invest..

[33] Zhao W., He X., Hoadley K.A. (2014). Comparison of RNA-Seq by poly (A) capture, ribosomal RNA depletion, and DNA microarray for expression profiling. BMC Genom..

[34] Deng X., Achari A., Federman S. (2020). Metagenomic sequencing with spiked primer enrichment for viral diagnostics and genomic surveillance. Nat. Microbiol..

[35] Shi Y., Wang G., Lau H.C., Yu J. (2022 Feb 16). Metagenomic sequencing for microbial DNA in human samples: emerging technological advances. Int. J. Mol. Sci..

[36] Bertrand D., Shaw J., Kalathiyappan M. (2019). Hybrid metagenomic assembly enables high-resolution analysis of resistance determinants and mobile elements in human microbiomes. Nat. Biotechnol..

[37] Kolmogorov M., Bickhart D.M., Behsaz B. (2020). metaFlye: scalable long-read metagenome assembly using repeat graphs. Nat. Methods.

[38] Pellow D., Zorea A., Probst M. (2021). SCAPP: an algorithm for improved plasmid assembly in metagenomes. Microbiome.

[39] Chen L., Zhao N., Cao J. (2022). Short- and long-read metagenomics expand individualized structural variations in gut microbiomes. Nat. Commun..

[40] Slizovskiy I.B., Oliva M., Settle J.K. (2022). Target-enriched long-read sequencing (TELSeq) contextualizes antimicrobial resistance genes in metagenomes. Microbiome.

[41] Ren Y., Chakraborty T., Doijad S., Falgenhauer L., Falgenhauer J., Goesmann A., Hauschild A.C., Schwengers O., Heider D. (2022 Jan 3). Prediction of antimicrobial resistance based on whole-genome sequencing and machine learning. Bioinformatics.

[42] Kim J.I., Maguire F., Tsang K.K., Gouliouris T., Peacock S.J., McAllister T.A., McArthur A.G., Beiko R.G. (2022 Sep 21). Machine learning for antimicrobial resistance prediction: current practice, limitations, and clinical perspective. Clin. Microbiol. Rev..

[43] Arango-Argoty G., Garner E., Pruden A., Heath L.S., Vikesland P., Zhang L. (2018 Feb 1). DeepARG: a deep learning approach for predicting antibiotic resistance genes from metagenomic data. Microbiome.

[44] McArthur A.G., Waglechner N., Nizam F., Yan A., Azad M.A., Baylay A.J., Bhullar K., Canova M.J., De Pascale G., Ejim L., Kalan L., King A.M., Koteva K., Morar M., Mulvey M.R., O'Brien J.S., Pawlowski A.C., Piddock L.J., Spanogiannopoulos P., Sutherland A.D., Tang I., Taylor P.L., Thaker M., Wang W., Yan M., Yu T., Wright G.D. (2013 Jul). The comprehensive antibiotic resistance database. Antimicrob. Agents Chemother..

[45] Florensa A.F., Kaas R.S., Clausen P.T.L.C., Aytan-Aktug D., Aarestrup F.M. (2022 Jan). ResFinder - an open online resource for identification of antimicrobial resistance genes in next-generation sequencing data and prediction of phenotypes from genotypes. Microb. Genom..

[46] Yang Y., Jiang X., Chai B., Ma L., Li B., Zhang A., Cole J.R., Tiedje J.M., Zhang T. (2016 Aug 1). ARGs-OAP: online analysis pipeline for antibiotic resistance genes detection from metagenomic data using an integrated structured ARG-database. Bioinformatics.

[47] Yin X., Jiang X.T., Chai B., Li L., Yang Y., Cole J.R., Tiedje J.M., Zhang T. (2018 Jul 1). ARGs-OAP v2.0 with an expanded SARG database and Hidden Markov Models for enhancement characterization and quantification of antibiotic resistance genes in environmental metagenomes. Bioinformatics.

[48] Gupta A., Kapil R., Dhakan D.B., Sharma V.K. (2014 Apr 15). MP3: a software tool for the prediction of pathogenic proteins in genomic and metagenomic data. PLoS One.

[49] Garg A., Gupta D. (2008 Jan 28). VirulentPred: a SVM based prediction method for virulent proteins in bacterial pathogens. BMC Bioinf..

[50] Sharma A., Garg A., Ramana J., Gupta D. (2023 Dec). VirulentPred 2.0: an improved method for prediction of virulent proteins in bacterial pathogens. Protein Sci..

[51] de Nies L., Lopes S., Busi S.B., Galata V., Heintz-Buschart A., Laczny C.C., May P., Wilmes P. (2021 Feb 17). PathoFact: a pipeline for the prediction of virulence factors and antimicrobial resistance genes in metagenomic data. Microbiome.

[52] Kuang X., Wang F., Hernandez K.M., Zhang Z., Grossman R.L. (2022 Feb 14). Accurate and rapid prediction of tuberculosis drug resistance from genome sequence data using traditional machine learning algorithms and CNN. Sci. Rep..

[53] Green A.G., Yoon C.H., Chen M.L., Ektefaie Y., Fina M., Freschi L., Gröschel M.I., Kohane I., Beam A., Farhat M. (2022 Jul 2). A convolutional neural network highlights mutations relevant to antimicrobial resistance in Mycobacterium tuberculosis. Nat. Commun..

[54] Planet P.J., Narechania A., Chen L., Mathema B., Boundy S., Archer G., Kreiswirth B. (2017 Feb). Architecture of a species: phylogenomics of Staphylococcus aureus. Trends Microbiol..

[55] Lakhundi S., Zhang K. (2018 Sep 12). Methicillin-resistant Staphylococcus aureus: molecular characterization, evolution, and epidemiology. Clin. Microbiol. Rev..

[56] Miragaia M. (2018 Nov 13). Factors contributing to the evolution of mecA-mediated β-lactam resistance in staphylococci: update and new insights from whole genome sequencing (WGS). Front. Microbiol..

[57] Eldholm V., Balloux F. (2016 Aug). Antimicrobial resistance in Mycobacterium tuberculosis: the odd one out. Trends Microbiol..

[58] Price V., Ngwira L.G., Lewis J.M., Baker K.S., Peacock S.J., Jauneikaite E., Feasey N. (2023 Feb). A systematic review of economic evaluations of whole-genome sequencing for the surveillance of bacterial pathogens. Microb. Genom..

[59] Abdelrahim S.S., Fouad M., Abdallah N., Ahmed R.F., Zaki S. (2021 Sep 29). Comparative study of CTX-M-15 producing Escherichia coli ST131 clone isolated from urinary tract infections and acute diarrhoea. Infect. Drug Resist..

[60] Popa L.I., Gheorghe I., Barbu I.C., Surleac M., Paraschiv S., Măruţescu L., Popa M., Pîrcălăbioru G.G., Talapan D., Niţă M., Streinu-Cercel A., Streinu-Cercel A., Oţelea D., Chifiriuc M.C. (2021 Jan 11). Multidrug resistant Klebsiella pneumoniae ST101 clone survival chain from inpatients to hospital effluent after chlorine treatment. Front. Microbiol..

[61] Rodrigues C., Lanza V.F., Peixe L., Coque T.M., Novais Â. (2023 Jun 15). Phylogenomics of globally spread clonal groups 14 and 15 of Klebsiella pneumoniae. Microbiol. Spectr..

[62] Avershina E., Shapovalova V., Shipulin G. (2021 Jul 21). Fighting antibiotic resistance in hospital-acquired infections: current state and emerging technologies in disease prevention, diagnostics and therapy. Front. Microbiol..

[63] Walsh T.R., Gales A.C., Laxminarayan R., Dodd P.C. (2023 Jul 3). Antimicrobial resistance: addressing a global threat to humanity. PLoS Med..

[64] Zhao J., He X., Min J., Yao R.S.Y., Chen Y., Chen Z., Huang Y., Zhu Z., Gong Y., Xie Y., Li Y., Luo W., Shi D., Xu J., Shen A., Wang Q., Sun R., He B., Lin Y., Shen N., Cao B., Yang L., She D., Shi Y., Zhou J., Su X., Zhou H., Ma Z., Fan H., Lin Y., Ye F., Nie X., Zhang Q., Tian X., Lai G., Zhou M., Ma J., Zhang J., Qu J. (2023 Oct). A multicenter prospective study of comprehensive metagenomic and transcriptomic signatures for predicting outcomes of patients with severe community-acquired pneumonia. EBioMedicine.

[65] Tran M., Smurthwaite K.S., Nghiem S., Cribb D.M., Zahedi A., Ferdinand A.D., Andersson P., Kirk M.D., Glass K., Lancsar E., AusPathoGen Program partners (2023 Nov). Economic evaluations of whole-genome sequencing for pathogen identification in public health surveillance and health-care-associated infections: a systematic review. Lancet Microbe.

[66] Baig S., Johannesen T.B., Overballe-Petersen S., Larsen J., Larsen A.R., Stegger M. (2018 Jul). Novel SCCmec type XIII (9A) identified in an ST152 methicillin-resistant Staphylococcus aureus. Infect. Genet. Evol..

[67] Maree M., Thi Nguyen L.T., Ohniwa R.L., Higashide M., Msadek T., Morikawa K. (2022 May 5). Natural transformation allows transfer of SCCmec-mediated methicillin resistance in Staphylococcus aureus biofilms. Nat. Commun..

[68] Shoji M., Cui L., Iizuka R., Komoto A., Neoh H.M., Watanabe Y., Hishinuma T., Hiramatsu K. (2011 Aug). walK and clpP mutations confer reduced vancomycin susceptibility in Staphylococcus aureus. Antimicrob. Agents Chemother..

[69] Kuroda M., Sekizuka T., Matsui H., Ohsuga J., Ohshima T., Hanaki H. (2019 Aug 14). IS256-Mediated overexpression of the WalKR two-component system regulon contributes to reduced vancomycin susceptibility in a Staphylococcus aureus clinical isolate. Front. Microbiol..

[70] Boinett C.J., Cain A.K., Hawkey J., Do Hoang N.T., Khanh N.N.T., Thanh D.P., Dordel J., Campbell J.I., Lan N.P.H., Mayho M., Langridge G.C., Hadfield J., Chau N.V.V., Thwaites G.E., Parkhill J., Thomson N.R., Holt K.E., Baker S. (2019 Feb). Clinical and laboratory-induced colistin-resistance mechanisms in Acinetobacter baumannii. Microb. Genom..

[71] Ramanathan B., Jindal H.M., Le C.F., Gudimella R., Anwar A., Razali R., Poole-Johnson J., Manikam R., Sekaran S.D. (2017 Aug 10). Next generation sequencing reveals the antibiotic resistant variants in the genome of Pseudomonas aeruginosa. PLoS One.

[72] Zhao S., Tyson G.H., Chen Y., Li C., Mukherjee S., Young S., Lam C., Folster J.P., Whichard J.M., McDermott P.F. (2015 Oct 30). Whole-genome sequencing analysis accurately predicts antimicrobial resistance phenotypes in Campylobacter spp. Appl. Environ. Microbiol..

[73] Roberts L.W., Harris P.N.A., Forde B.M., Ben Zakour N.L., Catchpoole E., Stanton-Cook M., Phan M.D., Sidjabat H.E., Bergh H., Heney C., Gawthorne J.A., Lipman J., Allworth A., Chan K.G., Chong T.M., Yin W.F., Schembri M.A., Paterson D.L., Beatson S.A. (2020). Integrating multiple genomic technologies to investigate an outbreak of carbapenemase-producing Enterobacter hormaechei. Nat. Commun..

[74] Roberts L.W., Forde B.M., Hurst T., Ling W., Nimmo G.R., Bergh H., George N., Hajkowicz K., McNamara J.F., Lipman J., Permana B., Schembri M.A., Paterson D., Beatson S.A., Harris P.N.A. (2021 Mar). Genomic surveillance, characterization and intervention of a polymicrobial multidrug-resistant outbreak in critical care. Microb. Genom..

[75] The CRyPTIC Consortium (2022 Aug 9). Genome-wide association studies of global Mycobacterium tuberculosis resistance to 13 antimicrobials in 10,228 genomes identify new resistance mechanisms. PLoS Biol..

[76] Farhat M.R., Freschi L., Calderon R., Ioerger T., Snyder M., Meehan C.J., de Jong B., Rigouts L., Sloutsky A., Kaur D., Sunyaev S., van Soolingen D., Shendure J., Sacchettini J., Murray M. (2019 May 13). GWAS for quantitative resistance phenotypes in Mycobacterium tuberculosis reveals resistance genes and regulatory regions. Nat. Commun..

[77] Naz S., Paritosh K., Sanyal P., Khan S., Singh Y., Varshney U., Nandicoori V.K. (2023 Jan 25). GWAS and functional studies suggest a role for altered DNA repair in the evolution of drug resistance in Mycobacterium tuberculosis. Elife.

[78] Ioerger T.R., Feng Y., Ganesula K., Chen X., Dobos K.M., Fortune S., Jacobs W.R., Mizrahi V., Parish T., Rubin E., Sassetti C., Sacchettini J.C. (2010 Jul). Variation among genome sequences of H37Rv strains of Mycobacterium tuberculosis from multiple laboratories. J. Bacteriol..

[79] Kavvas E.S., Catoiu E., Mih N., Yurkovich J.T., Seif Y., Dillon N., Heckmann D., Anand A., Yang L., Nizet V., Monk J.M., Palsson B.O. (2018 Oct 17). Machine learning and structural analysis of Mycobacterium tuberculosis pan-genome identifies genetic signatures of antibiotic resistance. Nat. Commun..

[80] Mortimer T.D., Zhang J.J., Ma K.C., Grad Y.H. (2022 May). Loci for prediction of penicillin and tetracycline susceptibility in Neisseria gonorrhoeae: a genome-wide association study. Lancet Microbe.

[81] Lamberte L.E., van Schaik W. (2022 Aug). Antibiotic resistance in the commensal human gut microbiota. Curr. Opin. Microbiol..

[82] Despotovic M., de Nies L., Busi S.B., Wilmes P. (2023 Jun). Reservoirs of antimicrobial resistance in the context of One Health. Curr. Opin. Microbiol..

[83] Hu Y., Yang X., Qin J., Lu N., Cheng G., Wu N., Pan Y., Li J., Zhu L., Wang X., Meng Z., Zhao F., Liu D., Ma J., Qin N., Xiang C., Xiao Y., Li L., Yang H., Wang J., Yang R., Gao G.F., Wang J., Zhu B. (2013). Metagenome-wide analysis of antibiotic resistance genes in a large cohort of human gut microbiota. Nat. Commun..

[84] Caselli E., Fabbri C., D'Accolti M., Soffritti I., Bassi C., Mazzacane S., Franchi M. (2020 May 18). Defining the oral microbiome by whole-genome sequencing and resistome analysis: the complexity of the healthy picture. BMC Microbiol..

[85] Kent A.G., Vill A.C., Shi Q., Satlin M.J., Brito I.L. (2020 Sep 1). Widespread transfer of mobile antibiotic resistance genes within individual gut microbiomes revealed through bacterial Hi-C. Nat. Commun..

[86] Forster S.C., Liu J., Kumar N., Gulliver E.L., Gould J.A., Escobar-Zepeda A., Mkandawire T., Pike L.J., Shao Y., Stares M.D., Browne H.P., Neville B.A., Lawley T.D. (2022 Mar 17). Strain-level characterization of broad host range mobile genetic elements transferring antibiotic resistance from the human microbiome. Nat. Commun..

[87] Wollein Waldetoft K., Sundius S., Kuske R., Brown S.P. (2023 Feb 28). Defining the benefits of antibiotic resistance in commensals and the scope for resistance optimization. mBio.

[88] Keith J.W., Pamer E.G. (2019 Jan 7). Enlisting commensal microbes to resist antibiotic-resistant pathogens. J. Exp. Med..

[89] Annavajhala M.K., Gomez-Simmonds A., Macesic N., Sullivan S.B., Kress A., Khan S.D., Giddins M.J., Stump S., Kim G.I., Narain R., Verna E.C., Uhlemann A.C. (2019 Oct 17). Colonizing multidrug-resistant bacteria and the longitudinal evolution of the intestinal microbiome after liver transplantation. Nat. Commun..

[90] Peng Z., Jin D., Kim H.B., Stratton C.W., Wu B., Tang Y.W., Sun X. (2017 Jul). Update on antimicrobial resistance in Clostridium difficile: resistance mechanisms and antimicrobial susceptibility testing. J. Clin. Microbiol..

[91] Weingarden A., González A., Vázquez-Baeza Y., Weiss S., Humphry G., Berg-Lyons D., Knights D., Unno T., Bobr A., Kang J., Khoruts A., Knight R., Sadowsky M.J. (2015 Mar 30). Dynamic changes in short- and long-term bacterial composition following fecal microbiota transplantation for recurrent Clostridium difficile infection. Microbiome.

[92] Langdon A., Schwartz D.J., Bulow C., Sun X., Hink T., Reske K.A., Jones C., Burnham C.D., Dubberke E.R., Dantas G., CDC Prevention Epicenter Program (2021 Feb 16). Microbiota restoration reduces antibiotic-resistant bacteria gut colonization in patients with recurrent Clostridioides difficile infection from the open-label PUNCH CD study. Genome Med..

[93] Bhattarai S.K., Du M., Zeamer A.L., M Morzfeld B., Kellogg T.D., Firat K., Benjamin A., Bean J.M., Zimmerman M., Mardi G., Vilbrun S.C., Walsh K.F., Fitzgerald D.W., Glickman M.S., Bucci V. (2024 Jan 17). Commensal antimicrobial resistance mediates microbiome resilience to antibiotic disruption. Sci. Transl. Med..

[94] Montassier E., Valdés-Mas R., Batard E., Zmora N., Dori-Bachash M., Suez J., Elinav E. (2021 Aug). Probiotics impact the antibiotic resistance gene reservoir along the human GI tract in a person-specific and antibiotic-dependent manner. Nat. Microbiol..

[95] van Duin D., Paterson D.L. (2016 Jun). Multidrug-resistant bacteria in the community: trends and lessons learned. Infect. Dis. Clin..

[96] Bui D.P., Chandran S.S., Oren E., Brown H.E., Harris R.B., Knight G.M., Grandjean L. (2021 Mar 18). Community transmission of multidrug-resistant tuberculosis is associated with activity space overlap in Lima, Peru. BMC Infect. Dis..

[97] Murase Y., Maeda S., Yamada H., Ohkado A., Chikamatsu K., Mizuno K., Kato S., Mitarai S. (2010 Jun). Clonal expansion of multidrug-resistant and extensively drug-resistant tuberculosis, Japan. Emerg. Infect. Dis..

[98] Shelenkov A., Petrova L., Zamyatin M., Mikhaylova Y., Akimkin V. (2021 Aug 20). Diversity of international high-risk clones of acinetobacter baumannii revealed in a Russian multidisciplinary medical center during 2017-2019. Antibiotics (Basel).

[99] Mazumder R., Hussain A., Abdullah A., Islam M.N., Sadique M.T., Muniruzzaman S.M., Tabassum A., Halim F., Akter N., Ahmed D., Mondal D. (2021 Oct 4). International high-risk clones among extended-spectrum β-lactamase-producing Escherichia coli in dhaka, Bangladesh. Front. Microbiol..

[100] Fuzi M., Rodriguez Baño J., Toth A. (2020 Feb 25). Global evolution of pathogenic bacteria with extensive use of fluoroquinolone agents. Front. Microbiol..

[101] Schmitz FJones ME., Hofmann B., Hansen B., Scheuring S., Lückefahr M., Fluit A., Verhoef J., Hadding U., Heinz H., Köhrer K. (1998). Characterization of grlA, grlB, gyrA, and gyrB mutations in 116 unrelated isolates of Staphylococcus aureus and effects of mutations on ciprofloxacin MIC. Antimicrob. Agents Chemother..

[102] Fuzi M., Szabo D., Csercsik R. (2017 Nov 16). Double-serine fluoroquinolone resistance mutations advance major international clones and lineages of various multi-drug resistant bacteria. Front. Microbiol..

[103] Loiseau C., Windels E.M., Gygli S.M., Jugheli L., Maghradze N., Brites D., Ross A., Goig G., Reinhard M., Borrell S., Trauner A., Dötsch A., Aspindzelashvili R., Denes R., Reither K., Beisel C., Tukvadze N., Avaliani Z., Stadler T., Gagneux S. (2023 Apr 8). The relative transmission fitness of multidrug-resistant Mycobacterium tuberculosis in a drug resistance hotspot. Nat. Commun..

[104] Hendriksen R.S., Munk P., Njage P., van Bunnik B., McNally L., Lukjancenko O., Röder T., Nieuwenhuijse D., Pedersen S.K., Kjeldgaard J., Kaas R.S., Clausen P.T.L.C., Vogt J.K., Leekitcharoenphon P., van de Schans M.G.M., Zuidema T., de Roda Husman A.M., Rasmussen S., Petersen B., Amid C., Cochrane G., Sicheritz-Ponten T., Schmitt H., Alvarez J.R.M., Aidara-Kane A., Pamp S.J., Lund O., Hald T., Woolhouse M., Koopmans M.P., Vigre H., Petersen T.N., Aarestrup F.M., Global Sewage Surveillance project consortium (2019 Mar 8). Global monitoring of antimicrobial resistance based on metagenomics analyses of urban sewage. Nat. Commun..

[105] Munk P., Brinch C., Møller F.D., Petersen T.N., Hendriksen R.S., Seyfarth A.M., Kjeldgaard J.S., Svendsen C.A., van Bunnik B., Berglund F., Larsson D.G.J., Koopmans M., Woolhouse M., Aarestrup F.M., Global Sewage Surveillance Consortium (2022 Dec 1). Genomic analysis of sewage from 101 countries reveals global landscape of antimicrobial resistance. Nat. Commun..

[106] Kirstahler P., Teudt F., Otani S., Aarestrup F.M., Pamp S.J. (2021 Jun 29). A peek into the plasmidome of global sewage. mSystems.

[107] Zhou Z., Shuai X., Lin Z., Yu X., Ba X., Holmes M.A., Xiao Y., Gu B., Chen H. (2023 Aug). Association between particulate matter (PM)2·5 air pollution and clinical antibiotic resistance: a global analysis. Lancet Planet. Health.

[108] Salyer S.J., Silver R., Simone K., Barton Behravesh C. (2017 Dec). Prioritizing zoonoses for global health capacity building-themes from one health zoonotic disease workshops in 7 countries, 2014-2016. Emerg. Infect. Dis..

[109] Govindaraj Vaithinathan A., Vanitha A. (2018). WHO global priority pathogens list on antibiotic resistance: an urgent need for action to integrate One Health data. Perspectives in Public Health.

[110] Van Boeckel T.P., Brower C., Gilbert M., Grenfell B.T., Levin S.A., Robinson T.P., Teillant A., Laxminarayan R. (2015 May 5). Global trends in antimicrobial use in food animals. Proc. Natl. Acad. Sci. U. S. A..

[111] He Y., Yuan Q., Mathieu J. (2020). Antibiotic resistance genes from livestock waste: occurrence, dissemination, and treatment. NPJ Clean Water.

[112] Waage J., Grace D., Fèvre E.M., McDermott J., Lines J., Wieland B., Naylor N.R., Hassell J.M., Chan K. (2022 Sep). Changing food systems and infectious disease risks in low-income and middle-income countries. Lancet Planet. Health.

[113] Bennani H., Mateus A., Mays N., Eastmure E., Stärk K.D.C., Häsler B. (2020 Jan 28). Overview of evidence of antimicrobial use and antimicrobial resistance in the food chain. Antibiotics (Basel).

[114] V T Nair D., Venkitanarayanan K., Kollanoor Johny A. (2018 Oct 11). Antibiotic-resistant Salmonella in the food supply and the potential role of antibiotic alternatives for control. Foods.

[115] Kipper D., Mascitti A.K., De Carli S., Carneiro A.M., Streck A.F., Fonseca A.S.K., Ikuta N., Lunge V.R. (2022 Aug 3). Emergence, dissemination and antimicrobial resistance of the main poultry-associated Salmonella serovars in Brazil. Vet. Sci..

[116] Samtiya M., Matthews K.R., Dhewa T., Puniya A.K. (2022 Sep 22). Antimicrobial resistance in the food chain: trends, mechanisms, pathways, and possible regulation strategies. Foods.

[117] Smith R.P., May H.E., AbuOun M., Stubberfield E., Gilson D., Chau K.K., Crook D.W., Shaw L.P., Read D.S., Stoesser N., Vilar M.J., Anjum M.F. (2023 Mar 14). A longitudinal study reveals persistence of antimicrobial resistance on livestock farms is not due to antimicrobial usage alone. Front. Microbiol..

[118] Zheng D., Yin G., Liu M., Hou L., Yang Y., Van Boeckel T.P., Zheng Y., Li Y. (2022 Nov 18). Global biogeography and projection of soil antibiotic resistance genes. Sci. Adv..

[119] Anthony W.E., Burnham C.D., Dantas G., Kwon J.H. (2021 Jun 16). The gut microbiome as a reservoir for antimicrobial resistance. J. Infect. Dis..

[120] Kelly S.A., Rodgers A.M., O'Brien S.C., Donnelly R.F., Gilmore B.F. (2020 Apr). Gut check time: antibiotic delivery strategies to reduce antimicrobial resistance. Trends Biotechnol..

[121] Yaffe E., Relman D.A. (2020 Feb). Tracking microbial evolution in the human gut using Hi-C reveals extensive horizontal gene transfer, persistence and adaptation. Nat. Microbiol..

[122] Grenfell B.T., Pybus O.G., Gog J.R., Wood J.L., Daly J.M., Mumford J.A., Holmes E.C. (2004 Jan 16). Unifying the epidemiological and evolutionary dynamics of pathogens. Science.

[123] Attwood S.W., Hill S.C., Aanensen D.M., Connor T.R., Pybus O.G. (2022 Sep). Phylogenetic and phylodynamic approaches to understanding and combating the early SARS-CoV-2 pandemic. Nat. Rev. Genet..

[124] Featherstone L.A., Zhang J.M., Vaughan T.G., Duchene S. (2022 Jun 2). Epidemiological inference from pathogen genomes: a review of phylodynamic models and applications. Virus Evol..

[125] Ingle D.J., Howden B.P., Duchene S. (2021 Sep). Development of phylodynamic methods for bacterial pathogens. Trends Microbiol..

[126] Rouli L., Merhej V., Fournier P.E., Raoult D. (2015 Jun 26). The bacterial pangenome as a new tool for analysing pathogenic bacteria. New Microbes New Infect..

[127] Kim Y., Gu C., Kim H.U., Lee S.Y. (2020 Jun). Current status of pan-genome analysis for pathogenic bacteria. Curr. Opin. Biotechnol..

[128] Bosi E., Fani R., Fondi M. (2015). Defining orthologs and pangenome size metrics. Methods Mol. Biol..

[129] von Meijenfeldt F.A.B., Hogeweg P., Dutilh B.E. (2023). A social niche breadth score reveals niche range strategies of generalists and specialists. Nat. Ecol. Evol..

[130] Baele G., Dellicour S., Suchard M.A., Lemey P., Vrancken B. (2018 Aug). Recent advances in computational phylodynamics. Curr. Opin. Virol..

[131] Frost S.D., Pybus O.G., Gog J.R., Viboud C., Bonhoeffer S., Bedford T. (2015 Mar). Eight challenges in phylodynamic inference. Epidemics.

[132] Li Y.F., Yang Y., Kong X.L., Song W.M., Li Y.M., Li Y.Y., Fang W.W., Yang J.Y., Men D., Yu C.B., Yang G.R., Han W.G., Liu W.Y., Yan K., Li H.C., Liu Y. (2024 Mar). Transmission dynamics and phylogeography of Mycobacterium tuberculosis in China based on whole-genome phylogenetic analysis. Int. J. Infect. Dis..

[133] Lapierre M., Blin C., Lambert A., Achaz G., Rocha E.P. (2016 Jul). The impact of selection, gene conversion, and biased sampling on the assessment of microbial demography. Mol. Biol. Evol..

[134] Vaughan T.G., Welch D., Drummond A.J., Biggs P.J., George T., French N.P. (2017 Feb). Inferring ancestral recombination graphs from bacterial genomic data. Genetics.

[135] Metcalf C.J., Birger R.B., Funk S., Kouyos R.D., Lloyd-Smith J.O., Jansen V.A. (2015 Mar). Five challenges in evolution and infectious diseases. Epidemics.

[136] Baquero F., Martínez J.L., F Lanza V., Rodríguez-Beltrán J., Galán J.C., San Millán A., Cantón R., Coque T.M. (2021 Dec 15). Evolutionary pathways and trajectories in antibiotic resistance. Clin. Microbiol. Rev..

[137] Gröschel M.I., Pérez-Llanos F.J., Diel R., Vargas Jr R., Escuyer V., Musser K., Lisa Trieu, Meissner J.S., Knorr J., Klinkenberg D., Kouw P., Homolka S., Samek W., Mathema B., Soolingen D.V., Niemann S., Ahuja S., Farhat M.R. (2022). Host-pathogen sympatry and differential transmissibility of Mycobacterium tuberculosis complex. medRxiv.

[138] Frazão N., Sousa A., Lässig M., Gordo I. (2019 Sep 3). Horizontal gene transfer overrides mutation in Escherichia coli colonizing the mammalian gut. Proc. Natl. Acad. Sci. U. S. A..

[139] Wang R., van Dorp L., Shaw L.P., Bradley P., Wang Q., Wang X., Jin L., Zhang Q., Liu Y., Rieux A., Dorai-Schneiders T., Weinert L.A., Iqbal Z., Didelot X., Wang H., Balloux F. (2018 Mar 21). The global distribution and spread of the mobilized colistin resistance gene mcr-1. Nat. Commun..

[140] Hyun J.C., Monk J.M., Szubin R., Hefner Y., Palsson B.O. (2023 Nov 24). Global pathogenomic analysis identifies known and candidate genetic antimicrobial resistance determinants in twelve species. Nat. Commun..

[141] Ali T., Ahmed S., Aslam M. (2023). Artificial intelligence for antimicrobial resistance prediction: challenges and opportunities towards practical implementation. Antibiotics.

[142] Rabaan A.A., Alhumaid S., Mutair A.A., Garout M., Abulhamayel Y., Halwani M.A., Alestad J.H., Bshabshe A.A., Sulaiman T., AlFonaisan M.K., Almusawi T., Albayat H., Alsaeed M., Alfaresi M., Alotaibi S., Alhashem Y.N., Temsah M.H., Ali U., Ahmed N. (2022 Jun 8). Application of artificial intelligence in combating high antimicrobial resistance rates. Antibiotics (Basel).

[143] Schrider D.R., Kern A.D. (2018 Apr). Supervised machine learning for population genetics: a new paradigm. Trends Genet..

[144] Uddin S., Khan A., Hossain M.E., Moni M.A. (2019 Dec 21). Comparing different supervised machine learning algorithms for disease prediction. BMC Med. Inf. Decis. Making.

[145] Maguire F., Jia B., Gray K.L., Lau W.Y.V., Beiko R.G., Brinkman F.S.L. (2020 Oct). Metagenome-assembled genome binning methods with short reads disproportionately fail for plasmids and genomic Islands. Microb. Genom..

[146] Aytan-Aktug D., Clausen P.T.L.C., Bortolaia V., Aarestrup F.M., Lund O. (2020 Jan 21). Prediction of acquired antimicrobial resistance for multiple bacterial species using neural networks. mSystems.

[147] Duarte A.S.R., Röder T., Van Gompel L., Petersen T.N., Hansen R.B., Hansen I.M., Bossers A., Aarestrup F.M., Wagenaar J.A., Hald T. (2021 Jan 15). Metagenomics-based approach to source-attribution of antimicrobial resistance determinants - identification of reservoir resistome signatures. Front. Microbiol..

[148] Deelder W., Napier G., Campino S., Palla L., Phelan J., Clark T.G. (2022 Jan 11). A modified decision tree approach to improve the prediction and mutation discovery for drug resistance in Mycobacterium tuberculosis. BMC Genom..

[149] Anahtar M.N., Yang J.H., Kanjilal S. (2021 Jun 18). Applications of machine learning to the problem of antimicrobial resistance: an emerging model for translational research. J. Clin. Microbiol..

[150] Ren Y., Chakraborty T., Doijad S., Falgenhauer L., Falgenhauer J., Goesmann A., Schwengers O., Heider D. (2022 Mar 10). Multi-label classification for multi-drug resistance prediction of Escherichia coli. Comput. Struct. Biotechnol. J..

[151] Ren Y., Chakraborty T., Doijad S., Falgenhauer L., Falgenhauer J., Goesmann A., Schwengers O., Heider D. (2022 Nov 12). Deep transfer learning enables robust prediction of antimicrobial resistance for novel. Antibiotics. Antibiotics (Basel).

[152] Hyun J.C., Kavvas E.S., Monk J.M., Palsson B.O. (2020 Mar 2). Machine learning with random subspace ensembles identifies antimicrobial resistance determinants from pan-genomes of three pathogens. PLoS Comput. Biol..

[153] Kaur J., Singh H., Sethi T. (2024 May 9). Emerging trends in antimicrobial resistance in bloodstream infections: multicentric longitudinal study in India (2017-2022). Lancet Reg Health Southeast Asia.

[154] Lapidus A.L., Korobeynikov A.I. (2021 Mar 23). Metagenomic data assembly - the way of decoding unknown microorganisms. Front. Microbiol..

[155] Setubal J.C. (2021 Nov 4). Metagenome-assembled genomes: concepts, analogies, and challenges. Biophys Rev.

[156] Benoit G., Raguideau S., James R., Phillippy A.M., Chikhi R., Quince C. (2023). Efficient High-Quality Metagenome Assembly from Long Accurate Reads using Minimizer-space de Bruijn Graphs. bioRxiv.

[157] Meyer F., Fritz A., Deng Z.L., Koslicki D., Lesker T.R., Gurevich A., Robertson G., Alser M., Antipov D., Beghini F., Bertrand D., Brito J.J., Brown C.T., Buchmann J., Buluç A., Chen B., Chikhi R., Clausen P.T.L.C., Cristian A., Dabrowski P.W., Darling A.E., Egan R., Eskin E., Georganas E., Goltsman E., Gray M.A., Hansen L.H., Hofmeyr S., Huang P., Irber L., Jia H., Jørgensen T.S., Kieser S.D., Klemetsen T., Kola A., Kolmogorov M., Korobeynikov A., Kwan J., LaPierre N., Lemaitre C., Li C., Limasset A., Malcher-Miranda F., Mangul S., Marcelino V.R., Marchet C., Marijon P., Meleshko D., Mende D.R., Milanese A., Nagarajan N., Nissen J., Nurk S., Oliker L., Paoli L., Peterlongo P., Piro V.C., Porter J.S., Rasmussen S., Rees E.R., Reinert K., Renard B., Robertsen E.M., Rosen G.L., Ruscheweyh H.J., Sarwal V., Segata N., Seiler E., Shi L., Sun F., Sunagawa S., Sørensen S.J., Thomas A., Tong C., Trajkovski M., Tremblay J., Uritskiy G., Vicedomini R., Wang Z., Wang Z., Wang Z., Warren A., Willassen N.P., Yelick K., You R., Zeller G., Zhao Z., Zhu S., Zhu J., Garrido-Oter R., Gastmeier P., Hacquard S., Häußler S., Khaledi A., Maechler F., Mesny F., Radutoiu S., Schulze-Lefert P., Smit N., Strowig T., Bremges A., Sczyrba A., McHardy A.C. (2022 Apr). Critical assessment of metagenome interpretation: the second round of challenges. Nat. Methods.

[158] Wu R., Davison M.R., Nelson W.C., Smith M.L., Lipton M.S., Jansson J.K., McClure R.S., McDermott J.E., Hofmockel K.S. (2023 Nov 23). Hi-C metagenome sequencing reveals soil phage-host interactions. Nat. Commun..

[159] Rand A.C., Jain M., Eizenga J.M., Musselman-Brown A., Olsen H.E., Akeson M., Paten B. (2017 Apr). Mapping DNA methylation with high-throughput nanopore sequencing. Nat. Methods.

[160] Tourancheau A., Mead E.A., Zhang X.S., Fang G. (2021 May). Discovering multiple types of DNA methylation from bacteria and microbiome using nanopore sequencing. Nat. Methods.

[161] Sczyrba A., Hofmann P., Belmann P., Koslicki D., Janssen S., Dröge J., Gregor I., Majda S., Fiedler J., Dahms E., Bremges A., Fritz A., Garrido-Oter R., Jørgensen T.S., Shapiro N., Blood P.D., Gurevich A., Bai Y., Turaev D., DeMaere M.Z., Chikhi R., Nagarajan N., Quince C., Meyer F., Balvočiūtė M., Hansen L.H., Sørensen S.J., Chia B.K.H., Denis B., Froula J.L., Wang Z., Egan R., Don Kang D., Cook J.J., Deltel C., Beckstette M., Lemaitre C., Peterlongo P., Rizk G., Lavenier D., Wu Y.W., Singer S.W., Jain C., Strous M., Klingenberg H., Meinicke P., Barton M.D., Lingner T., Lin H.H., Liao Y.C., Silva G.G.Z., Cuevas D.A., Edwards R.A., Saha S., Piro V.C., Renard B.Y., Pop M., Klenk H.P., Göker M., Kyrpides N.C., Woyke T., Vorholt J.A., Schulze-Lefert P., Rubin E.M., Darling A.E., Rattei T., McHardy A.C. (2017 Nov). Critical assessment of metagenome interpretation-a benchmark of metagenomics software. Nat. Methods.

[162] Rebelo A.R., Bortolaia V., Leekitcharoenphon P., Hansen D.S., Nielsen H.L., Ellermann-Eriksen S., Kemp M., Røder B.L., Frimodt-Møller N., Søndergaard T.S., Coia J.E., Østergaard C., Westh H., Aarestrup F.M. (2022 Jun 10). One day in Denmark: comparison of phenotypic and genotypic antimicrobial susceptibility testing in bacterial isolates from clinical settings. Front. Microbiol..

[163] Papp M., Solymosi N. (2022 Mar 4). Review and comparison of antimicrobial resistance gene databases. Antibiotics (basel). Erratum in: Antibiotics (Basel).

[164] Léger A., Lambraki I., Graells T., Cousins M., Henriksson P.J.G., Harbarth S., Carson C.A., Majowicz S.E., Troell M., Parmley E.J., Jørgensen P.S., Wernli D. (2021 Aug 26). Characterizing social-ecological context and success factors of antimicrobial resistance interventions across the One Health spectrum: analysis of 42 interventions targeting E. coli. BMC Infect. Dis..

[165] Bottery M.J., Pitchford J.W., Friman V.P. (2021 Apr). Ecology and evolution of antimicrobial resistance in bacterial communities. ISME J..

[166] Oliver A., Xue Z., Villanueva Y.T., Durbin-Johnson B., Alkan Z., Taft D.H., Liu J., Korf I., Laugero K.D., Stephensen C.B., Mills D.A., Kable M.E., Lemay D.G. (2022 Jun 28). Association of diet and antimicrobial resistance in healthy U.S. Adults. mBio.

[167] Radovanovic M., Kekic D., Gajic I., Kabic J., Jovicevic M., Kekic N., Opavski N., Ranin L. (2023 Feb 1). Potential influence of antimicrobial resistance gene content in probiotic bacteria on the gut resistome ecosystems. Front. Nutr..

[168] Njage P.M.K., van Bunnik B., Munk P., Marques A.R.P., Aarestrup F.M. (2023 Nov). Association of health, nutrition, and socioeconomic variables with global antimicrobial resistance: a modelling study. Lancet Planet. Health.

[169] Li J., Xie S., Ahmed S., Wang F., Gu Y., Zhang C., Chai X., Wu Y., Cai J., Cheng G. (2017 Jun 13). Antimicrobial activity and resistance: influencing factors. Front. Pharmacol..

[170] Cunha B.A. (2016). An infectious disease and pharmacokinetic perspective on oral antibiotic treatment of uncomplicated urinary tract infections due to multidrug-resistant Gram-negative uropathogens: the importance of urinary antibiotic concentrations and urinary pH. Eur. J. Clin. Microbiol. Infect. Dis..

[171] Singleton D.A., Pongchaikul P., Smith S., Bengtsson R.J., Baker K., Timofte D., Steen S., Jones M., Roberts L., Sánchez-Vizcaíno F., Dawson S., Noble P.M., Radford A.D., Pinchbeck G.L., Williams N.J. (2021 Jul 30). Temporal, spatial, and genomic analyses of Enterobacteriaceae clinical antimicrobial resistance in companion animals reveals phenotypes and genotypes of one health concern. Front. Microbiol..

[172] Gheorghe-Barbu I., Barbu I.C., Popa L.I., Pîrcălăbioru G.G., Popa M., Măruțescu L., Niță-Lazar M., Banciu A., Stoica C., Gheorghe Ș., Lucaciu I., Săndulescu O., Paraschiv S., Surleac M., Talapan D., Muntean A.A., Preda M., Muntean M.M., Dragomirescu C.C., Popa M.I., Oțelea D., Chifiriuc M.C. (2022 Sep 14). Temporo-spatial variations in resistance determinants and clonality of Acinetobacter baumannii and Pseudomonas aeruginosa strains from Romanian hospitals and wastewaters. Antimicrob. Resist. Infect. Control.

[173] Fleming-Davies A.E., Williams P.D., Dhondt A.A., Dobson A.P., Hochachka W.M., Leon A.E., Ley D.H., Osnas E.E., Hawley D.M. (2018 Mar 2). Incomplete host immunity favors the evolution of virulence in an emergent pathogen. Science.

[174] Micoli F., Bagnoli F., Rappuoli R., Serruto D. (2021 May). The role of vaccines in combatting antimicrobial resistance. Nat. Rev. Microbiol..

[175] Kuchina A., Brettner L.M., Paleologu L., Roco C.M., Rosenberg A.B., Carignano A., Kibler R., Hirano M., DePaolo R.W., Seelig G. (2021 Feb 19). Microbial single-cell RNA sequencing by split-pool barcoding. Science.

[176] McNulty R., Sritharan D., Pahng S.H., Meisch J.P., Liu S., Brennan M.A., Saxer G., Hormoz S., Rosenthal A.Z. (2023 May). Probe-based bacterial single-cell RNA sequencing predicts toxin regulation. Nat. Microbiol..

[177] Wang B., Lin A.E., Yuan J., Novak K.E., Koch M.D., Wingreen N.S., Adamson B., Gitai Z. (2023 Aug 31). Single-cell massively-parallel multiplexed microbial sequencing (M3-seq) identifies rare bacterial populations and profiles phage infection. Nat. Microbiol..

[178] Sherry N.L., Horan K.A., Ballard S.A., Gonҫalves da Silva A., Gorrie C.L., Schultz M.B., Stevens K., Valcanis M., Sait M.L., Stinear T.P., Howden B.P., Seemann T. (2023 Jan 4). An ISO-certified genomics workflow for identification and surveillance of antimicrobial resistance. Nat. Commun..

[179] Mendes I., Griffiths E., Manuele A., Fornika D., Tausch S., Le-Viet T. (2024). hAMRonization: enhancing antimicrobial resistance prediction using the PHA4GE AMR detection specification and tooling. bioRxiv.

[180] Palmer G.H., Buckley G.J., National Academies of Sciences (2021). Combating Antimicrobial Resistance and Protecting the Miracle of Modern Medicine.

[181] Robillard D.W., Sundermann A.J., Raux B.R., Prinzi A.M. (2024 Apr 19). Navigating the network: a narrative overview of AMR surveillance and data flow in the United States. Antimicrob. Steward Healthc. Epidemiol..

[182] Vong S., Anciaux A., Hulth A., Stelling J., Thamlikitkul V., Gupta S., Fuks J.M., Walia K., Rattanumpawan P., Eremin S., Tisocki K., Sedai T.R., Sharma A. (2017 Sep 5). Using information technology to improve surveillance of antimicrobial resistance in South East Asia. BMJ.

[183] Taaffe J., Sharma R., Parthiban A.B.R., Singh J., Kaur P., Singh B.B., Gill J.P.S., Gopal D.R., Dhand N.K., Parekh F.K. (2023 Mar 7). One Health activities to reinforce intersectoral coordination at local levels in India. Front. Public Health.

[184] Jackson R.W., Johnson L.J., Clarke S.R., Arnold D.L. (2011 Jan). Bacterial pathogen evolution: breaking news. Trends Genet..

[185] Bonneaud C., Longdon B. (2020 Sep 3). Emerging pathogen evolution: using evolutionary theory to understand the fate of novel infectious pathogens. EMBO Rep..

[186] Sironi M., Cagliani R., Forni D., Clerici M. (2015 Apr). Evolutionary insights into host-pathogen interactions from mammalian sequence data. Nat. Rev. Genet..

[187] Georgieva M., Buckee C.O., Lipsitch M. (2019 Jan). Models of immune selection for multi-locus antigenic diversity of pathogens. Nat. Rev. Immunol..

[188] Rothenburg S., Brennan G. (2020 Jan). Species-specific host-virus interactions: implications for viral host range and virulence. Trends Microbiol..

[189] Matic I. (2019 Aug 8). Mutation rate heterogeneity increases odds of survival in unpredictable environments. Mol. Cell..

[190] Ebert D., Fields P.D. (2020 Dec). Host-parasite co-evolution and its genomic signature. Nat. Rev. Genet..

[191] Obeng N., Bansept F., Sieber M., Traulsen A., Schulenburg H. (2021 Sep). Evolution of microbiota-host associations: the microbe's perspective. Trends Microbiol..

[192] Rayan R.A. (2023 Feb 26). Flare of the silent pandemic in the era of the COVID-19 pandemic: obstacles and opportunities. World J. Clin. Cases.

[193] D'Costa V.M., King C.E., Kalan L., Morar M., Sung W.W., Schwarz C., Froese D., Zazula G., Calmels F., Debruyne R., Golding G.B., Poinar H.N., Wright G.D. (2011 Aug 31). Antibiotic resistance is ancient. Nature.

[194] Peterson E., Kaur P. (2018 Nov 30). Antibiotic resistance mechanisms in bacteria: relationships between resistance determinants of antibiotic producers, environmental bacteria, and clinical pathogens. Front. Microbiol..

[195] Diaz Caballero J., Wheatley R.M., Kapel N., López-Causapé C., Van der Schalk T., Quinn A., Shaw L.P., Ogunlana L., Recanatini C., Xavier B.B., Timbermont L., Kluytmans J., Ruzin A., Esser M., Malhotra-Kumar S., Oliver A., MacLean R.C. (2023 Jul 12). Mixed strain pathogen populations accelerate the evolution of antibiotic resistance in patients. Nat. Commun..

[196] Moura de Sousa J., Balbontín R., Durão P., Gordo I. (2017 Apr 18). Multidrug-resistant bacteria compensate for the epistasis between resistances. PLoS Biol..

[197] Durão P., Balbontín R., Gordo I. (2018 Aug). Evolutionary mechanisms shaping the maintenance of antibiotic resistance. Trends Microbiol..

[198] Munita J.M., Arias C.A. (2016 Apr). Mechanisms of antibiotic resistance. Microbiol. Spectr..

[199] Balasubramanian D., López-Pérez M., Grant T.A., Ogbunugafor C.B., Almagro-Moreno S. (2022 Sep). Molecular mechanisms and drivers of pathogen emergence. Trends Microbiol..

[200] Darby E.M., Trampari E., Siasat P., Gaya M.S., Alav I., Webber M.A., Blair J.M.A. (2023). Molecular mechanisms of antibiotic resistance revisited. Nat. Rev. Microbiol..

[201] Baindara P., Ghosh A.K., Mandal S.M. (2020 Aug). Coevolution of resistance against antimicrobial peptides. Microb. Drug Resist..

[202] Gong T., Fu J., Shi L., Chen X., Zong X. (2021 Sep 30). Antimicrobial peptides in gut health: a review. Front. Nutr..

[203] Ramirez J., Guarner F., Bustos Fernandez L., Maruy A., Sdepanian V.L., Cohen H. (2020 Nov 24). Antibiotics as major disruptors of gut microbiota. Front. Cell. Infect. Microbiol..

[204] Baindara P., Mandal S.M. (2023 Dec 14). Gut-antimicrobial peptides: synergistic Co-evolution with antibiotics to combat multi-antibiotic resistance. Antibiotics (Basel).

[205] Costa F., Teixeira C., Gomes P., Martins M.C.L. (2019). Clinical application of AMPs. Adv. Exp. Med. Biol..

[206] De Oliveira D.M.P., Forde B.M., Kidd T.J., Harris P.N.A., Schembri M.A., Beatson S.A., Paterson D.L., Walker M.J. (2020 May 13). Antimicrobial resistance in ESKAPE pathogens. Clin. Microbiol. Rev..

[207] Seung K.J., Keshavjee S., Rich M.L. (2015 Apr 27). Multidrug-resistant tuberculosis and extensively drug-resistant. Tuberculosis. Cold Spring Harb Perspect Med..

[208] Papanicolas L.E., Gordon D.L., Wesselingh S.L., Rogers G.B. (2018 May). Not just antibiotics: is cancer chemotherapy driving antimicrobial resistance?. Trends Microbiol..

[209] Villemin C., Six A., Neville B.A., Lawley T.D., Robinson M.J., Bakdash G. (2023 Jan). The heightened importance of the microbiome in cancer immunotherapy. Trends Immunol..

[210] Wang Y., Jenq R.R., Wargo J.A., Watowich S.S. (2023 Mar 6). Microbiome influencers of checkpoint blockade-associated toxicity. J. Exp. Med..

[211] Nanayakkara A.K., Boucher H.W., Fowler V.G., Jezek A., Outterson K., Greenberg D.E. (2021 Nov). Antibiotic resistance in the patient with cancer: escalating challenges and paths forward. Ca - Cancer J. Clin..

[212] Danielsen A.S., Franconeri L., Page S., Myhre A.E., Tornes R.A., Kacelnik O., Bjørnholt J.V. (2023 Apr 18). Clinical outcomes of antimicrobial resistance in cancer patients: a systematic review of multivariable models. BMC Infect. Dis..

[213] Wang Y., Yu Z., Ding P., Lu J., Mao L., Ngiam L., Yuan Z., Engelstädter J., Schembri M.A., Guo J. (2023 Jan 31). Antidepressants can induce mutation and enhance persistence toward multiple antibiotics. Proc. Natl. Acad. Sci. U. S. A..

[214] Lu J., Ding P., Wang Y. (2022). Antidepressants promote the spread of extracellular antibiotic resistance genes via transformation. ISME Commun..

[215] Li M.C., Lu J., Lu Y., Xiao T.Y., Liu H.C., Lin S.Q., Xu D., Li G.L., Zhao X.Q., Liu Z.G., Zhao L.L., Wan K.L. (2021 Oct 5). rpoB mutations and effects on rifampin resistance in Mycobacterium tuberculosis. Infect. Drug Resist..

[216] Du D., Wang-Kan X., Neuberger A., van Veen H.W., Pos K.M., Piddock L.J.V., Luisi B.F. (2018 Sep). Multidrug efflux pumps: structure, function and regulation. Nat. Rev. Microbiol..

[217] Remm S., Earp J.C., Dick T., Dartois V., Seeger M.A. (2022 Feb 9). Critical discussion on drug efflux in Mycobacterium tuberculosis. FEMS Microbiol. Rev..

[218] Nasiri M.J., Haeili M., Ghazi M., Goudarzi H., Pormohammad A., Imani Fooladi A.A., Feizabadi M.M. (2017 Apr 25). New insights in to the intrinsic and acquired drug resistance mechanisms in mycobacteria. Front. Microbiol..

[219] Daniel C., Bhakta S. (2022 May). Immunobiology of tubercle bacilli and prospects of immunomodulatory drugs to tackle tuberculosis (TB) and other non-tubercular mycobacterial infections. Immunobiology.

[220] Laws M., Jin P., Rahman K.M. (2022 Jan). Efflux pumps in Mycobacterium tuberculosis and their inhibition to tackle antimicrobial resistance. Trends Microbiol..

[221] Brauner A., Fridman O., Gefen O., Balaban N.Q. (2016 Apr). Distinguishing between resistance, tolerance and persistence to antibiotic treatment. Nat. Rev. Microbiol..

[222] Crabbé A., Jensen P.Ø., Bjarnsholt T., Coenye T. (2019 Oct). Antimicrobial tolerance and metabolic adaptations in microbial biofilms. Trends Microbiol..

[223] Ragheb M.N., Thomason M.K., Hsu C., Nugent P., Gage J., Samadpour A.N., Kariisa A., Merrikh C.N., Miller S.I., Sherman D.R., Merrikh H. (2019 Jan 3). Inhibiting the evolution of antibiotic resistance. Mol. Cell..

[224] Merrikh H., Kohli R.M. (2020 Oct). Targeting evolution to inhibit antibiotic resistance. FEBS J..

[225] Al Mamun A.A., Lombardo M.J., Shee C., Lisewski A.M., Gonzalez C., Lin D., Nehring R.B., Saint-Ruf C., Gibson J.L., Frisch R.L., Lichtarge O., Hastings P.J., Rosenberg S.M. (2012 Dec 7). Identity and function of a large gene network underlying mutagenic repair of DNA breaks. Science.

[226] Schrader S.M., Vaubourgeix J., Nathan C. (2020 Jun 24). Biology of antimicrobial resistance and approaches to combat it. Sci. Transl. Med..

[227] Pribis J.P., Zhai Y., Hastings P.J., Rosenberg S.M. (2022 Jun 28). Stress-induced mutagenesis, gambler cells, and stealth targeting antibiotic-induced evolution. mBio.

[228] Bhatnagar K., Wong A. (2019 Nov 5). The mutational landscape of quinolone resistance in Escherichia coli. PLoS One.

[229] Revitt-Mills S.A., Robinson A. (2020 Oct 22). Antibiotic-induced mutagenesis: under the microscope. Front. Microbiol..

[230] Zhai Y., Pribis J.P., Dooling S.W., Garcia-Villada L., Minnick P.J., Xia J., Liu J., Mei Q., Fitzgerald D.M., Herman C., Hastings P.J., Costa-Mattioli M., Rosenberg S.M. (2023 Jun 23). Drugging evolution of antibiotic resistance at a regulatory network hub. Sci. Adv..

[231] Rehman A., Patrick W.M., Lamont I.L. (2019 Jan). Mechanisms of ciprofloxacin resistance in Pseudomonas aeruginosa: new approaches to an old problem. J. Med. Microbiol..

[232] Raymond B. (2019 May 14). Five rules for resistance management in the antibiotic apocalypse, a road map for integrated microbial management. Evol. Appl..

[233] Wright G.D. (2016 Nov). Antibiotic adjuvants: rescuing antibiotics from resistance. Trends Microbiol..

[234] Kumar V., Yasmeen N., Pandey A., Ahmad Chaudhary A., Alawam A.S., Ahmad Rudayni H., Islam A., Lakhawat S.S., Sharma P.K., Shahid M. (2023 Dec 20). Antibiotic adjuvants: synergistic tool to combat multi-drug resistant pathogens. Front. Cell. Infect. Microbiol..

[235] Bernal P., Molina-Santiago C., Daddaoua A., Llamas M.A. (2013 Sep). Antibiotic adjuvants: identification and clinical use. Microb. Biotechnol..

[236] Plackett B. (2022 Dec). Three ways to combat antimicrobial resistance. Nature.

[237] Hitchcock N.M., Devequi Gomes Nunes D., Shiach J., Valeria Saraiva Hodel K., Dantas Viana Barbosa J., Alencar Pereira Rodrigues L., Coler B.S., Botelho Pereira Soares M., Badaró R. (2023 Apr 21). Current clinical landscape and global potential of bacteriophage therapy. Viruses.

[238] Lin D.M., Koskella B., Lin H.C. (2017 Aug 6). Phage therapy: an alternative to antibiotics in the age of multi-drug resistance. World J. Gastrointest. Pharmacol. Therapeut.

[239] Hill V., Ruis C., Bajaj S., Pybus O.G., Kraemer M.U.G. (2021 Dec). Progress and challenges in virus genomic epidemiology. Trends Parasitol..

[240] Gire S.K., Goba A., Andersen K.G. (2014 Sep 12). Genomic surveillance elucidates Ebola virus origin and transmission during the 2014 outbreak. Science.

[241] Dudas G., Carvalho L.M., Bedford T. (2017 Apr 20). Virus genomes reveal factors that spread and sustained the Ebola epidemic. Nature.

[242] Di Paola N., Sanchez-Lockhart M., Zeng X., Kuhn J.H., Palacios G. (2020). Viral genomics in Ebola virus research. Nat. Rev. Microbiol..

[243] Dhar M.S., Marwal R., Vs R., Ponnusamy K., Jolly B., Bhoyar R.C., Sardana V., Naushin S., Rophina M., Mellan T.A., Mishra S., Whittaker C., Fatihi S., Datta M., Singh P., Sharma U., Ujjainiya R., Bhatheja N., Divakar M.K., Singh M.K., Imran M., Senthivel V., Maurya R., Jha N., Mehta P., Sharma P., Vr A., Chaudhary U., Soni N., Thukral L., Flaxman S., Bhatt S., Pandey R., Dash D., Faruq M., Lall H., Gogia H., Madan P., Kulkarni S., Chauhan H., Sengupta S., Kabra S., Gupta R.K., Singh S.K., Agrawal A., Rakshit P., Nandicoori V., Tallapaka K.B., Sowpati D.T., Thangaraj K., Bashyam M.D., Dalal A., Sivasubbu S., Scaria V., Parida A., Raghav S.K., Prasad P., Sarin A., Mayor S., Ramakrishnan U., Palakodeti D., Seshasayee A.S.N., Bhat M., Shouche Y., Pillai A., Dikid T., Das S., Maitra A., Chinnaswamy S., Biswas N.K., Desai A.S., Pattabiraman C., Manjunatha M.V., Mani R.S., Arunachal Udupi G., Abraham P., Atul P.V., Cherian S.S., Indian SARS-CoV-2 Genomics Consortium (INSACOG)‡ (2021 Nov 19). Genomic characterization and epidemiology of an emerging SARS-CoV-2 variant in Delhi, India. Science.

[244] Kanteh A., Jallow H.S., Manneh J., Sanyang B., Kujabi M.A., Ndure S.L., Jarju S., Sey A.P., Damilare K.D., Bah Y., Sambou S., Jarju G., Manjang B., Jagne A., Bittaye S.O., Bittaye M., Forrest K., Tiruneh D.A., Samateh A.L., Jagne S., Hué S., Mohammed N., Amambua-Ngwa A., Kampmann B., D'Alessandro U., de Silva T.I., Roca A., Sesay A.K. (2023 Mar). Genomic epidemiology of SARS-CoV-2 infections in the Gambia: an analysis of routinely collected surveillance data between March, 2020, and January, 2022. Lancet Global Health.

[245] Cárdenas P., Corredor V., Santos-Vega M. (2022 Jul 15). Genomic epidemiological models describe pathogen evolution across fitness valleys. Sci. Adv..

[246] Duval A., Opatowski L., Brisse S. (2023 May). Defining genomic epidemiology thresholds for common-source bacterial outbreaks: a modelling study. Lancet Microbe.

